# Model-based action planning involves cortico-cerebellar and basal ganglia networks

**DOI:** 10.1038/srep31378

**Published:** 2016-08-19

**Authors:** Alan S. R. Fermin, Takehiko Yoshida, Junichiro Yoshimoto, Makoto Ito, Saori C. Tanaka, Kenji Doya

**Affiliations:** 1 Graduate School of Information Science, Nara Institute of Science and Technology, Nara 630-0192, Japan; 2 Neural Computation Unit, Okinawa Institute of Science and Technology Graduate University, Okinawa 904-0495, Japan; 3 Brain Science Institute, Tamagawa University, Tokyo 194-8610, Japan; 4 ATR Brain Information Communication Research Lab, Kyoto 619-0288, Japan

## Abstract

Humans can select actions by learning, planning, or retrieving motor memories. Reinforcement Learning (RL) associates these processes with three major classes of strategies for action selection: exploratory RL learns state-action values by exploration, model-based RL uses internal models to simulate future states reached by hypothetical actions, and motor-memory RL selects past successful state-action mapping. In order to investigate the neural substrates that implement these strategies, we conducted a functional magnetic resonance imaging (fMRI) experiment while humans performed a sequential action selection task under conditions that promoted the use of a specific RL strategy. The ventromedial prefrontal cortex and ventral striatum increased activity in the exploratory condition; the dorsolateral prefrontal cortex, dorsomedial striatum, and lateral cerebellum in the model-based condition; and the supplementary motor area, putamen, and anterior cerebellum in the motor-memory condition. These findings suggest that a distinct prefrontal-basal ganglia and cerebellar network implements the model-based RL action selection strategy.

Using exploration and reward feedback, humans and other animals have a remarkable capacity to learn new motor behaviors without explicit teaching^1^. Throughout most of our lives, however, we depend on explicit or implicit knowledge, based upon past experiences, such as a map of the area or properties of the musculoskeletal system, to enable focused exploration and efficient learning^2^,^3^. After repeated practice, a motor behavior becomes stereotyped and can be executed with little mental load^4^. What brain mechanisms enable animals to employ different learning strategies and to select or integrate them in a given situation? In this paper, we take a new behavioral paradigm that captures different stages of motor learning during a single experimental session^5^, and using fMRI we explore brain structures that are specifically involved in implementing different learning strategies.

The theory of reinforcement learning (RL)^6^ prescribes three major classes of algorithms for action selection and learning: motor-memory, exploratory, and model-based strategies. The motor-memory strategy reinforces the sequence of states and actions that led to successful results in past experiences, which is simple, but requires many trials before finding an optimal sequence, unless there are clues to minimize exploration. The exploratory strategy recursively updates values of states and actions to efficiently utilize experiences resulting from exploratory actions, acquired rewards, and state transitions. The model-based strategy employs an internal model that enables simulation of the future state reached by a hypothetical action, or multiple actions. Since these strategies require different degrees of pre-acquired knowledge and computational loads for real-time execution, it is reasonable to speculate that humans may utilize them depending on their experience level with a certain context or task.

Computational models of RL and fMRI studies with humans have explored the neural substrates of model-based and motor-memory strategies, given their strong resemblance to classical, psychological, dichotomous behavior control employing deliberative and automatic processes, respectively. Activity in the dorsolateral prefrontal cortex (DLPFC) has been associated with the use of model-based strategies when an internal model of environmental dynamics is available and can be used for forward planning and prediction of an action’s future outcomes^7^,^8^,^9^,^10^. Conversely, activation of the posterior dorsal striatum is observed when actions become automatic after extensive practice, and a motor-memory strategy is more likely to control behavior^10^,^11^. However, accumulating behavioral evidence^4^,^5^,^12^,^13^, supported by computational models of learning and decision-making^14^,^15^, suggests that humans may use multiple decision strategies for learning, including exploratory, model-based, and motor-memory strategies. Furthermore, based on anatomical^16^,^17^,^18^,^19^, electrophysiological and lesion studies with monkeys^12^,^20^ and fMRI studies with humans learning complex sequential behaviors^12^,^21^,^22^, it has been suggested that such strategies are implemented in the brain by parallel anatomical neural networks linking different regions of the PFC, striatum, and cerebellum^12^,^14^,^23^,^24^. Yet, it remains unclear whether and when humans use multiple selection strategies, and whether such strategies are implemented in local or larger brain networks.

Our working hypothesis is that humans rely on distinct action selection strategies for learning, depending on their level of experience with a task: (1) in the early stage of learning with no prior knowledge of state transition or reward setting, the exploratory strategy is used; (2) as learning continues and an internal model of action results is developed, the model-based strategy is used to expedite learning; (3) in the late stage, after many successful experiences, a motor-memory strategy is used for robust performance with minimal computational load. In order to test this hypothesis and to identify brain mechanisms supporting different action selection strategies, we designed a task paradigm, the “grid-sailing task^5^”, which recreates different stages of motor learning during a single session inside an fMRI scanner.

The grid-sailing task requires a subject to move a cursor from its start position to a goal position in a 5 × 5 grid by performing a sequence of key presses with the right index, middle, and ring fingers (Fig. 1). Different cursor shapes representing key-mapping (KM) rules were combined with different start-goal (SG) positions (Fig. 1b). What makes the task interesting is that the cursor can move in only three of eight neighbor positions, such that reaching the goal requires zigzag movement of the cursor (Fig. S1). This favors model-based, multi-step planning of key presses and cursor movements over habitual or exploratory behavior. Subjects performed this task under three task conditions designed to mimic specific learning stages. In Condition 1, subjects learned new action sequences with unknown KM rules; in Condition 2, subjects used pre-learned KMs to learn new action sequences, and in Condition 3 subjects performed well-learned action sequences. Our working hypothesis predicted that the absence of an internal model in Condition 1 would favor the use of an exploratory strategy for action learning. In Condition 2, the availability of internal models of the KMs would promote the use of a model-based strategy for forward planning. In Condition 3, after repeated successful experiences, a motor-memory strategy would be employed because it requires less computation in real time and allows prompt, efficient execution of stored sequential motor responses.

We asked subjects to perform each task condition either with or without a delay after a KM and SG positions were presented. We expected that subjects would use this pre-start delay period for mental simulation of imaginary actions if an internal model of the KM rule was available. We examined behavioral measures to test our hypotheses on differential use of action selection strategies and analyzed fMRI signals especially during the pre-start delay period, to identify neural networks associated with behavior suggestive of the use of model-based action planning.

## Results

**Figure 1:**
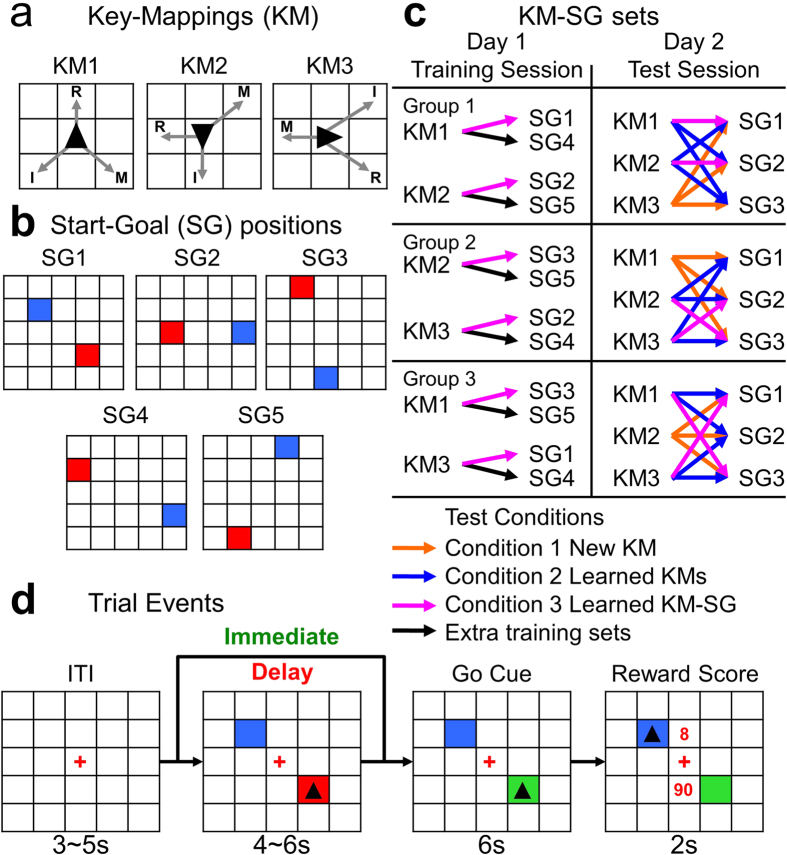
Grid-sailing task and experimental design. (**a**) Key-mappings (KM) and respective right hand fingers (I: index; M: middle; R: ring). Arrows indicate the three directions of motion depending on the current position of the cursor. (**b**) Pairs of Start-Goal (SG) positions used in training and test sessions. Start position (red) and goal position (blue). (**c**) KM-SG sets assigned for practice in training and test sessions for each group of subjects. Colored arrows in the training session: magenta – KM-SG sets chosen for retest in the test session; black arrows – extra KM-SG sets for training. Colored arrows in the test session: orange – KM-SG sets for test Condition 1, blue – KM-SG sets for test Condition 2, and magenta – KM-SG sets for test Condition 3. (**d**) Task sequence flow. The task started with presentation of a 5 × 5 grid and a fixation cross (FC) for 3 s~5 s. Next, a pair of SG positions was displayed. A cursor (triangle) representing one specific KM was simultaneously displayed on top of the start position. The color of the start position indicated whether the subject’s response would start immediately (green) or after a delay (red) of 4 s~6 s. In trials with a delay, when the color switched from red to green, subjects had 6 s to perform the task. The subject’s task was to execute a sequence of finger movements to move the cursor to the goal position by the shortest pathway. Performance feedback, with the number of moves and the reward score, was displayed for 2 s at the end of the trial, above and below the FC, respectively, after which the next trial restarted, following a variable inter-trial interval.

Subjects (n = 18) performed the “grid-sailing task”^5^ on two consecutive days (Fig. 1c). The goal of the task was to move a cursor from its start position to a target goal position on a 5 × 5 grid via the shortest possible path. A trial started with presentation of a pair of SG positions and the cursor located on the start position. The cursor shape represented the KM used in the trial (Fig. 1a). SG positions were chosen so that there was no straight path from the start to the goal using either KM. See Fig. S1 for examples of paths learned by subjects. The color of the start position specified the start time condition: green for an immediate start or red for a delayed start when subjects had to withhold a response for 4 ~ 6 s until the start position turned green (Fig. 1d). If a subject could successfully reach the goal position within 6 s, a reward score of 100 points, minus 5 points for each excessive key press deviating from the shortest path was presented. If the subject did not reach the goal a reward score of 0 points was presented.

In the training session on day 1, subjects trained with two of the KMs paired with two SGs each (Fig. 1c). The KM-SG combinations used on day 1 were counter-balanced across three subject groups. Subjects practiced the four KM-SG combinations randomly, in alternate short blocks of immediate or delayed starts, in a total of 60 trials evenly distributed in three trial blocks (see Supplementary Methods). In the last five trials of the third block, the success rate was more than 70% with an average reward score of more than 80 points for every subject, and the sample mean of the reward score was 95 points (Fig. S2 and Supplementary Methods for a detailed analysis of performance behavior in the training session). This analysis suggests that subjects were well acquainted with the KMs and learned fixed action sequences for each trained KM-SG combination, although the question of whether they established internal models for those KMs had to be tested by using novel SGs in the test session.

During the test session on day 2 (Fig. 1c), subjects performed combinations of KMs (the two KMs learned in the training session and a newly introduced KM) and SG positions (the two SGs used in the training session and a new SG), resulting in 3 × 3 KM-SG combinations. Each of the nine resulting KM-SG combinations was performed with immediate and delayed starts for 20 trials (see Supplementary Methods). The KM-SG combinations comprised three task conditions (Fig. 1c): Condition 1 was a combination of a novel KM with three SG positions; Condition 2 was a combination of the learned KMs with new SG positions; and Condition 3 was the KM-SG combinations learned in the training session.

### Behavior performance in the fMRI test session

**Figure 2:**
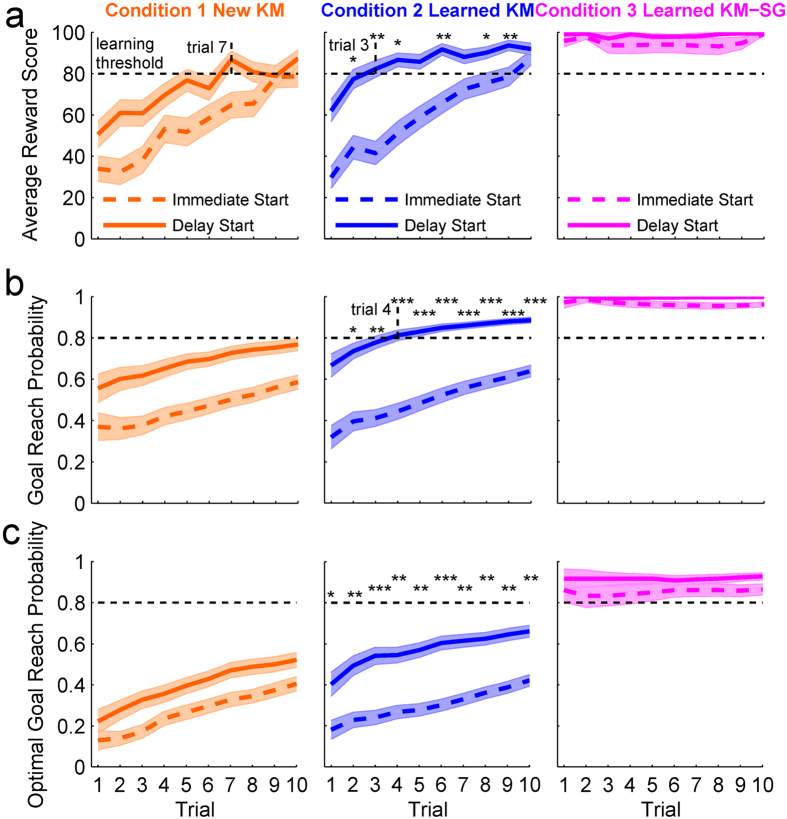
Trial-by-trial analysis of behavior performance in the test session shows the benefit of pre-start delay with pre-learned key-maps in Condition 2. (**a**) Average reward score. In Condition 1 the average reward score reached the learning criteria only in trial 7, whereas it was reached in trial 3 in Condition 2. Stars on top of each trial in Condition 2 represent the statistical significance against the same trial in Condition 1, for performances in trials with the delayed start. The same notation appears in Fig. 2b and Fig. 2c. (**b**) Goal reach probability. This is a measure of successful goal reach. In this analysis, the learning criterion (80% accuracy) was not reached in Condition 1, whereas in Condition 2 the criterion was reached in trial 4. (**c**) Optimal goal reach probability was the probability of reaching the goal by performing the shortest sequence of finger movements. All trials following a delay period in Condition 2 are statistically significant compared to the same trials in Condition 1. Shaded areas represent the standard error.

One important prediction based on our hypothesis is that the use of a distinct action selection strategy in each task condition would be associated with a distinct behavioral performance. The lack of prior experience and of an internal model due to the introduction of a new KM in Condition 1 would be associated with slow learning and a lower reward score relative to performance in Condition 2, where the presence of an internal model of the KMs would lead to fast learning and higher reward score, especially in trials preceded by a delay when the internal model of a KM could be used for forward planning. In Condition 3 we expected a stable, fast, accurate performance for the pre-learned action sequences. In support of this prediction, we found a significant effect of task condition (F_(2,3204)_ = 186.54, p < 0.0001), with a significantly (p < 0.001) lower overall reward score in Condition 1 (mean = 64.03, SE = 1.08) than in Condition 2 (mean = 72.77, SE = 0.94) and the largest reward score in Condition 3 (mean = 96.77, SE = 1.33) (See Fig. S3 and Supplementary Methods for statistical analysis). A trial-by-trial analysis revealed that subjects learned faster in trials with a pre-start delay and achieved the learning criteria (80% accuracy in reward score) earlier in Condition 2 (criteria achieved in trial 3, Fig. 2a middle panel) than in Condition 1 (criteria achieved in trial 7, Fig. 2a left panel). Across early and later trial blocks, significant increases in reward scores were observed in Condition 1 and Condition 2, but not in Condition 3 (Fig. S4 and S5). Reward scores in Condition 1 and Condition 2 were significantly larger in trials with a delay than in immediate start trials (p < 0.0001), but no such effect was observed in Condition 3 (p = 0.86). Furthermore, the reward score in delayed-start trials was significantly larger in Condition 2 than in Condition 1 (p < 0.0001), whereas no significant difference was found for performances in immediate start trials between these conditions (p > 0.05). We did not find a significant effect of subject group, F_(2,3204)_ = 2.74, p = 0.0649, indicating that the effect of task condition, trial block, and start time was comparable among groups.

To further investigate the effect of the availability of an internal model of KMs on behavior performance, we estimated subjects’ overall trial-by-trial probability of reaching the goal (goal reach probability) and the probability of reaching the goal by performing the shortest action sequence (optimal goal reach probability). Analysis of the goal reach probability found that subjects could reach a learning criterion of 80% success at trial 4 in delayed-start trials in Condition 2 (versus trial 4 in Condition 1, p < 0.001, Fig. 2b middle panel), but failed to achieve the learning criterion in Condition 1 (Fig. 2b left panel). The analysis of optimal goal reach probability found a significant difference between Conditions 1 and 2, which emerged in the first trial (p < 0.05) and remained significant throughout the entire experimental session (Fig. 2c).

**Figure 3:**
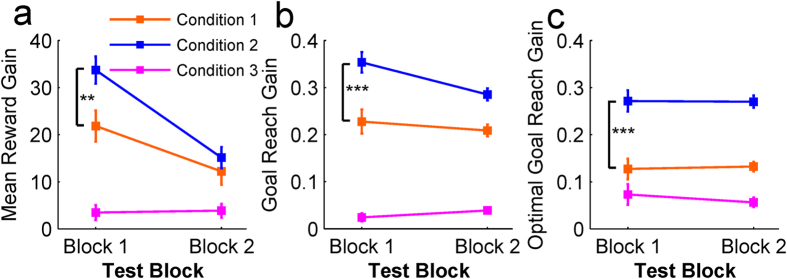
Performance gains with a delayed start were significantly larger in Condition 2. Performance gain is the difference of a specific behavior measure between trials with and without a delay. Significant differences between Condition 1 and Condition 2 were found for (**a**) Mean Reward Gain, (**b**) Goal Reach Gain, and (**c**) Optimal Goal Reach Gain. Error bars represent standard errors.

The beneficial effect of a delayed start on sequential action planning was further analyzed by calculating the reward gain, defined as the difference between the reward score of the delay and the immediate start trials for each KM-SG set. A large, positive reward gain indicates that subjects were able to perform more successful action sequences in pre-start delay trials than in immediate start trials. The reward gain in Condition 2 was larger than in Condition 1, especially during the first trial block (p < 0.01) (Fig. 3a). We also found a beneficial effect of delayed start in Condition 2 relative to Condition 1, when the performance gain was computed using the goal reach probability (Fig. 3b) and optimal goal reach probability (Fig. 3c). These performance improvements are consistent with our hypothesis that internal models of KM in Condition 2 were used for sequential action planning during the pre-start delay period.

In order to further quantify performance differences among the task conditions, we classified trials into three trial types based on reward score subjects received at the end of each trial: error trials (reward score = 0; failed to reach the goal), suboptimal trials (0< reward score <100; reached the goal by a longer path), and optimal trials (reward score = 100; reached the goal by an optimal path; See Supplementary Methods for detailed statistical results). We expected to find a higher rate of error trials in Condition 1 relative to Condition 2 due to the lack of experience with the new KM. Conversely, we expected higher rate of optimal trials in Condition 2 given that the internal model of KMs could be useful for successful action planning. As for Condition 3, we expected to find the lowest rate of error trials and the highest rate of optimal trials, given the well-learned action sequences. These predictions were supported by the analysis, which revealed a significant effect of task condition (p < 0.0001), with a higher percentage of optimal trials in Condition 2 than in Condition 1 (p < 0.006), especially in delayed start trials (p < 0.01; Fig. S8). The percentage of error trials was significantly lower in Condition 2 (p < 0.001) and in Condition 3 (p < 0.0001) than in Condition 1 (Fig. S8). Furthermore, the percentage of error trials under the delayed-start condition was not significantly different between Conditions 2 and 3 (p > 0.4; Fig. S8).

In order to identify the possible cause of the high error rate in Condition 1, we estimated the mean absolute deviation (MAD) as a measure of exploratory behavior. MAD was estimated separately for reward score, the number of steps to reach the goal, and execution time, and entered into a group analysis with a three-way ANOVA (See Supplementary Methods for further statistical results). This analysis revealed higher performance variability in reward score, number of steps, and action sequence execution time in Condition 1, than in Condition 2 (p < 0.001) or 3 (p < 0.001). These results suggest that the high error rate and variable performance in Condition 1 may be explained by active action exploration and learning of the new KM.

These results show that subject performance in terms of reward score, overall goal reach and optimal goal reach were improved quite significantly in Condition 2, specifically with a delayed start, a finding that replicates our previous work^5^. This is a strong indication that subjects had acquired internal models of KMs in the training session and used them during the pre-start delay period in Condition 2 for assessment of hypothetical key-press sequences and resulting cursor paths.

## Neuroimaging

### Delay-period Activity in Conditions 1 and 2

**Figure 4:**
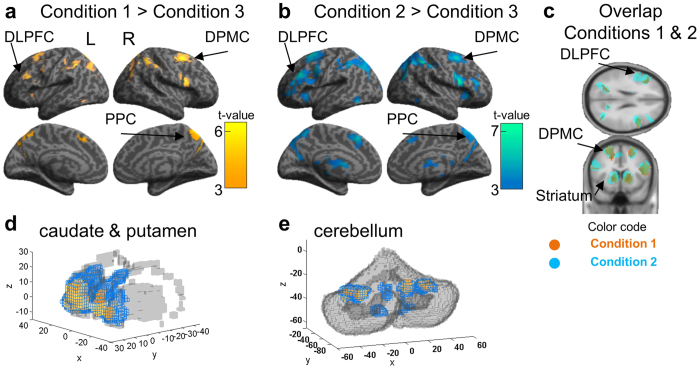
Delay-period BOLD signal was most extensive in Condition 2 in the cerebral cortex, the basal ganglia, and the cerebellum. Cortical and subcortical areas showing increased delay-period BOLD signal in Conditions 1 and 2 using activity during the delay-period in Condition 3 as a control. (**a**) Delay-period activation in Condition 1 versus Condition 3. (**b**) Delay-period activation in Condition 2 versus Condition 3. This analysis revealed a similar recruitment of brain areas in both Conditions 1 and 2, including the dorsolateral prefrontal cortex, lateral and dorsal premotor cortices and posterior parietal cortex. (**c**) Overlapping activation in Condition 1 and Condition 2 (images displayed in **a** and **b)** demonstrates broader activation in Condition 2. (**d**) Basal-ganglia (caudate and putamen)-extracted voxels from the comparison between Condition 1 and Condition 3 (orange voxels) and Condition 2 and Condition 3 (blue voxels). (**e**) Cerebellum-extracted voxels in the same way as in **d**. As in cortical areas, the extent of activity in the basal ganglia and cerebellum was larger in Condition 2 than in Condition 1. The axis in **d** and **e** represents the MNI coordinates in millimeters.

Our main purpose was to identify the neural network that accomplishes mental simulation of sequential actions using internal models. Due to the beneficial effect of the pre-start delay in accelerating learning in Condition 2 when subjects could use the internal model of the KMs to plan sequential actions, we analyzed how the BOLD fMRI signal in the delay period was modulated by different task conditions. Using Condition 3 as the control task for Condition 1 (Condition 1 > Condition 3) and for Condition 2 (Condition 2 > Condition 3), the analysis revealed that both Condition 1 (Fig. 4a) and Condition 2 (Fig. 4b) activated similar brain areas, including the left DLPFC, dorsal and lateral premotor cortices, parietal cortices, striatum, and cerebellum bilaterally (p < 0.05, FWE corrected, Table S1). Extension of this activation was larger in Condition 2 than in Condition 1 in cortical areas (Fig. 4c), in the striatum (Fig. 4d) and cerebellum (4e). In the striatum the number of activated voxels in Condition 2 (left BG = 242 voxels, peak voxel T-value: 6.28; right BG = 249 voxels, peak voxel T-value: 6.04) was much larger than in Condition 1 (left BG = 130, peak voxel T-value: 5.9; right BG = 71, peak voxel T-value: 5.7).

**Figure 5:**
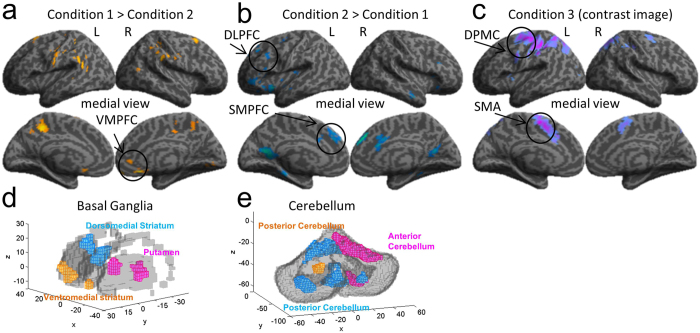
Comparison of the delay-period BOLD signal shows signatures of model-free, model-based, and memory-based action selection strategies. (**a**) Brain regions in Condition 1 (subtraction: Condition 1 – Condition 2) that we associate with the use of trial-based strategies, showing significantly increased activation in the right ventromedial prefrontal cortex, left somatosensory cortex, angular gyrus, posterior cingulate, and ventromedial striatum. (**b**) Regions in Condition 2 (subtraction: Condition 2 – Condition 1) associated with the model-based strategy, revealed activity in the dorsolateral prefrontal cortex, anterior cingulate cortex, superior medial prefrontal cortex, ventrolateral premotor cortex, ventral precuneus and dorsomedial striatum. (**c**) Delay-period neural activity in Condition 3 (positive contrast image of Condition 3) was found in the proper-supplementary motor area, motor cortex, left putamen, and right anterior cerebellum. (**d**) Striatum voxel activation clusters extracted from the results in (**a**), (**b**) and (**c**). Condition 1: orange clusters; Condition 2: blue clusters; Condition 3: pink clusters. (**e**) Cerebellum voxel activation clusters extracted from the results in (**a**), (**b**) and (**c**). Condition 1: orange clusters; Condition 2: blue clusters; Condition 3: pink clusters. The x, y and z axes in d and e represent MNI coordinates in millimeters.

In order to investigate whether distinct neural networks were associated with the distinct performance profiles found in the behavior analysis, we conducted a direct contrast of the delay period signal between Conditions 1 and 2 (Condition 1 > Condition 2; Condition 2 > Condition1). Cortical activation in Condition 1 was found in the ventromedial orbital frontal cortex (VMOFC), the somatosensory cortex, the inferior parietal lobule, and primary visual cortex (Fig. 5a, p < 0.05, corrected). Contrastingly, cortical activation in Condition 2 was found in the left DLPFC, ventral premotor cortex bilaterally, and right inferior temporal gyrus (Fig. 5b, p < 0.05, corrected). Using anatomical mask images we extracted the voxels activated in the striatum and cerebellum. In Condition 1 significant activation was found in the anterior-ventromedial part of the striatum (peak voxel: x = −12, y = 20, z = −5, Fig. 5d yellow voxel clusters; p < 0.05, corrected for the mask image). Conversely, striatal activation in Condition 2 was found in a dorsomedial region (caudate body, peak voxel: x = 9, y = 2, z = 16; Fig. 5d blue voxel clusters; p < 0.05, corrected for the mask image). Furthermore, lateral parts of the posterior cerebellum were also active in Condition 2 (Fig. 5e).

### Delay-period Activity in Condition 3

We also investigated neural mechanisms associated with well-learned action sequences in Condition 3. When contrasting the activity of Condition 3 versus the activities in Condition 1, Condition 2 or both, no significant activation was found (p > 0.05, corrected). However, when analyzing the positive activation (contrast image) of Condition 3 we found significant activity in the dorsal premotor cortex, the supplementary motor area (SMA), and the superior parietal cortex (Fig. 5c; p < 0.05, corrected). Activation was also found in the dorsal striatum (putamen region, Fig. 5d, p < 0.05, corrected for the mask image) and right anterior cerebellum (Fig. 5e, p < 0.05, corrected for the mask image).

### BOLD Signal Change in the Delay-period

**Figure 6:**
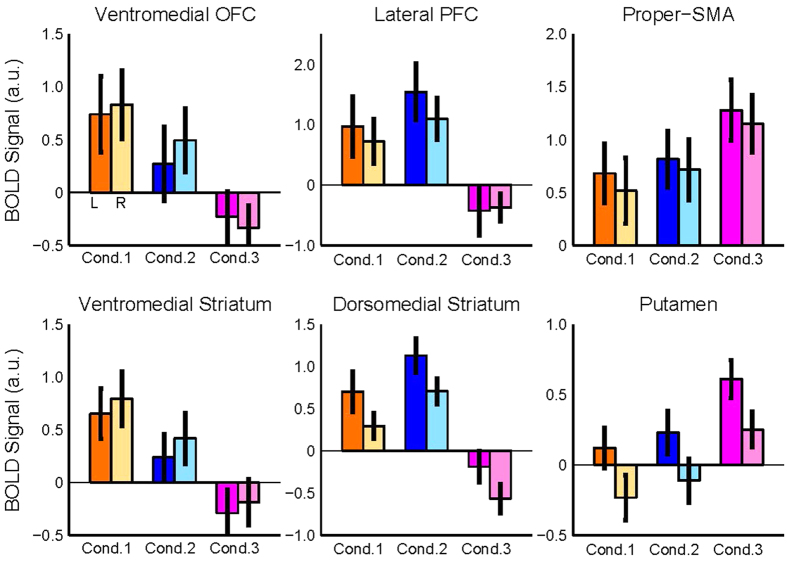
Distinct cortico-basal ganglia circuits recruited in each task condition, mimicking different learning phases. Plots show the BOLD signal intensity (beta parameters), separated for each task condition and cerebral hemispheres (left and right bars) in the analysis presented in Fig. 5 The signal for Condition 1 was estimated from the VMOFC and ventral striatum, for Condition 2 from the DLPFC and dorsomedial striatum and the signal for Condition 3 from the proper-SMA and putamen.

The above fMRI analyses revealed activation of distinct prefrontal, basal ganglia, and cerebellar regions in each task condition during the pre-start delay. In order to further characterize activity in these regions, we estimated the fMRI signal (beta parameters) in the identified prefrontal and striatal clusters (See Supplementary Methods for detailed analysis). Signal intensity in the VMOFC and the anterior-ventromedial striatum was highest in Condition 1 (Fig. 6, leftmost top and bottom panels). Conversely, signal intensity in the DLPFC and dorsomedial striatum was highest in Condition 2 (Fig. 6, middle top and bottom panels). Finally, in Condition 3 the highest signal intensity was found in the SMA and putamen (Fig. 6, rightmost top and bottom panels).

### Performance-period Activity

**Figure 7:**
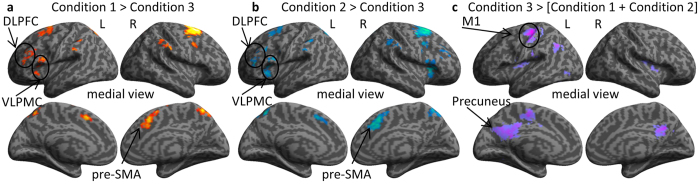
BOLD signal during the movement performance period showed distinct activities for newly learned and well-learned actions. (**a**) Brain signal during the performance period in Condition 1 versus the performance period in Condition 3. (**b**) Brain signal during the performance period in Condition 2 versus the performance period in Condition 3. (**c**) Brain signal during the performance period in Condition 3 versus the summation of the performance period in Condition 1 and Condition 2. Results presented in **a**, **b**, and **c** reflect brain signals in the performance periods of delay-start trials.

Unlike activity during the delay period, brain activity during the performance period can be a mixture of processes that reflect action planning, motor execution, and learning from the results of actions. A direct contrast between Conditions 1 and 2 did not find any significant difference in their activities during the performance periods of immediate or pre-start delay trials (p < 0.05, uncorrected) or even when using a more lenient statistical threshold of p < 0.001, uncorrected. However, using Condition 3 as the control, significant activation was found in Condition 1 (Condition 1 - Condition 3, Fig. 7a) and in Condition 2 (Condition 2 – Condition 3, Fig. 7b) in the left DLPFC, the bilateral ventral premotor cortices, the pre-SMA, the bilateral inferior parietal cortices, and the superior precuneus. Interestingly, we also found activities in Condition 2 in the right dorsolateral prefrontal cortex and the right posterior cerebellum. A contrast between Condition 3 and Condition 1 with Condition 2 (Condition 3 – [Condition 1 + Condition 2]) showed significant activity in areas related to automatic behavior control such as the left somatosensory cortex, left motor cortices, left SMA, left inferior parietal cortex, bilateral ventral precuneus, bilateral posterior putamen, and the anterior cerebellum (Fig. 7c).

### Reward-modulated Activity

**Figure 8:**
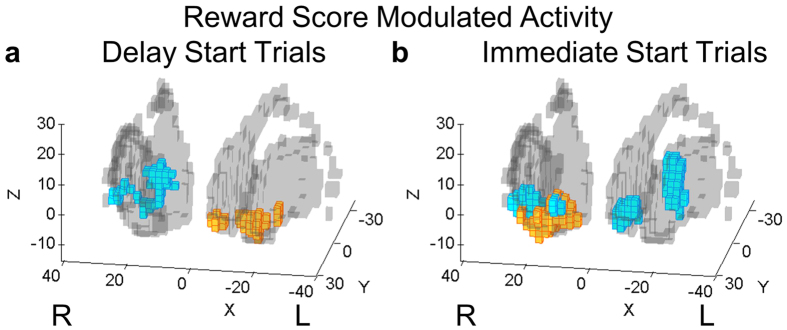
Reward score-modulated activities in the basal ganglia differed according to the start time condition. The reward score earned at the end of each trial was used as a parametric modulator of the reward score display period, separated for each test condition and pooled across the two scanning sessions (p < 0.001, uncorrected). Reward score modulated activity in Condition 1 (yellow voxels) was found in the ventromedial striatum, in the ventromedial and dorsomedial striatum in Condition 2 (blue voxels), and ventromedial and lateral putamen in Condition 3 (magenta voxels). The axes in **a** and **b** represent MNI coordinates in millimeters.

We also performed a parametric modulation analysis between BOLD signals in the striatum and reward scores during the feedback period. Delayed-start and immediate start trials were analyzed separately (Table S4). In Condition 1, activation in delayed-start trials was found in the anterior-medial region of the left caudate nucleus (T-value: 3.39) (Fig. 8a, yellow voxels), whereas in immediate-start trials, activation was found in the same region, but on the right hemisphere (T-value: 6.06) (Fig. 8b, yellow voxels). Activation in Condition 2 in delayed trials was found in the medial (caudate) and lateral (putamen) regions of the right striatum (peak T-value: 4.28) (Fig. 8a, blue voxels), and bilaterally in the putamen and caudate regions of the striatum in trials without the delay (peak T-value: 8.09) (Fig. 8b, blue voxels).

## Discusssion

In order to clarify what action strategies are used in different stages of learning and to identify the brain networks implementing those strategies, we performed an fMRI experiment using the grid-sailing task and showed that the behavioral results were in general agreement with our hypothesis and replicated our previous findings using the same behavioral paradigm^5^. In Condition 1, with no initial knowledge of the KM, learning was slow and erratic, and performance was highly variable. This behavioral profile suggests the likely use of an exploratory strategy. In Condition 2, prior experience with the KM facilitated learning with new goals, especially when a pre-start delay was available. This finding indicates that the model-based strategy is likely to have been employed using the learned model of the KM for forward planning of the path toward the goal and for corresponding action sequences. The stable, accurate, and rapid performance in Condition 3 suggests use of a motor-memory strategy to reproduce pre-learned action sequences. Our simplistic prediction was that the performance advantage from the pre-start delay time would be possible only when the internal model of the KM was available, i.e., in Conditions 2 and 3. However, the significant advantage of the pre-start delay in Condition 1 suggests that internal models can also be acquired and utilized even in the early stage of learning. Another possibility is that there is a general advantage to having pre-start preparation time, which is not specific to the use of internal models. This may explain performance differences in very early trials in Condition 1, when the use of an internal model was highly unlikely.

The exploratory behavior in Condition 1, evidenced by high performance variability, was associated with activation of the VMOFC and anterior ventral-medial striatum during the delay period (Fig. 5a). Previous brain imaging studies revealed a role of the VMOFC in valuation of goods and complex cues^25^, in learning action-outcome associations^26^,^27^ and implicated the ventral striatum in state evaluation (critic)^28^. The significant activation of both the VMPFC and ventral striatum in Condition 1 and their association with stimulus and action value learning suggest that an exploratory algorithm with value function learning, such as the actor-critic algorithm, may have been used in Condition 1. This interpretation is further supported by a significant activation in Condition 1 of the ventral part of the anterior prefrontal cortex (APFC, x = 9, y = 56, z = −17, Fig. 5a). Hierarchical models of prefrontal function identify the APFC as a coordinator of multiple processes, such as cognitive branching^29^, decision monitoring^30^, relational integration, and construction of representations^31^. A model-based fMRI study also found a strong correlation between APFC activity and exploratory action selection^32^. Activation in the APFC, VMPFC and ventral striatum have also been found in initial stages of goal-directed learning^33^ and sequential action learning by trial-and-error, when exploratory action selection plays a major role in learning^21^,^22^. We speculate that APFC activity in Condition 1 may have contributed to learning the model of the new KM by creating a representation between button press and a cursor motion direction through action exploration and integration of action-outcome value signals estimated in the VMPFC and striatum during on-line action exploration.

An important contribution of the present study is the introduction of the pre-start delay, which allows the use of internal models if available. When a pre-start delay was available, a significant increase in accuracy in generating optimal action sequences in the very first trial was observed in Condition 2 (Fig. 2c), a finding consistent with that of a previous study using a two-step decision-making task^8^. Performance improvements in Condition 2 were associated with activity during the delay in the DLPFC, ventrolateral premotor cortex, pre-SMA, dorsomedial striatum, and lateral cerebellum. The DLPFC and ventral premotor cortex have been implicated in mental imagery and model-based decision making^7^,^8^,^10^,^14^,^34^,^35^. The posterior lateral cerebellum and the dorsomedial striatum have loop connections with the DLPFC^16^,^17^,^18^,^19^,^36^. It has been suggested that the lateral cerebellum models the body and the environment^14^,^37^,^38^,^39^. Activities of dorsomedial striatal neurons represent values of actions being selected^40^,^41^. These observations are consistent with the hypothesis that the cerebellar internal model predicts the resulting state from a hypothetical candidate action, the prefrontal cortex holds it as its working memory, and the striatum evaluates the goodness of the predicted state for selection or rejection of the candidate action^14^.

The deliberative role of the prefrontal cortex in learning and model-based decision-making has been demonstrated by sequential learning and two-step decision-making tasks^8^,^21^,^22^,^35^,^42^,^43^. The activations of the VMPFC in Condition 1 and DLPFC in Condition 2 are in general agreement with the role of the prefrontal cortex in deliberative control. However, prefrontal activation in different locations and the distinct performance profiles associated with Conditions 1 and 2 suggest the use of deliberation for different purposes. The highly erratic and exploratory behavior in Condition 1 suggests that use of a model-based strategy as expected in Condition 2, where optimal sequence planning and accuracy following the delay period were significantly higher from the very first trial, is unlikely. The significant activation of the DLPFC in Condition 2 and substantial performance improvements when the internal model of KMs could be used for sequential action planning provide support for the hypothesis that the DLPFC is also involved in multi-step sequential action planning^14^. This hypothesis is further supported by a neuroimaging study with humans showing involvement of the DLPFC in a path-planning task^44^ and by electrophysiological recordings in monkeys showing that DLPFC activity codes sequences of immediate and future behavioral goals^45^.

Analysis of the BOLD signal in the striatum in the delay and feedback periods suggests a possible differentiation of its functions in action valuation and reward-based learning as demonstrated in previous works^28^,^40^,^41^,^46^. Neuroimaging studies strongly point to the role of the ventral striatum in reward-based learning^28^,^33^,^46^ and in exploration of novel actions in unfamiliar and uncertain environments^47^. The finding that striatal activity in Condition 1 was located predominantly in the ventral anterior striatum during both delay and feedback periods provides further evidence that action selection and learning in this condition were guided primarily by exploration and reward feedback. Conversely, in Condition 2 delay activity was found in the dorsomedial striatum and feedback period activity was found in the anterior putamen and extended into the ventral striatum. Given the anatomical connections between the DLPFC and dorsal striatum^16^,^18^,^19^,^36^,^48^ we speculate that the dorsomedial caudate may evaluate the goodness of predictions generated in the DLPFC-cerebellum network during the delay period, whereas activity in the anterior putamen and the ventral striatum may evaluate the quality of the executed actions based on actual feedback so that successful sequence of actions would be reinforced for future reuse.

High accuracy and fast reaction time in Condition 3 (Fig. 2, Figs S3 and S7) were associated with activity in the SMA, posterior putamen, and right anterior cerebellum during the delay period. Activity in the SMA, putamen, and anterior cerebellum increases significantly in advanced stages of motor learning when actions become automatic and are generated with little cognitive and attentional effort^10^,^12^,^21^,^22^,^23^,^24^,^49^. The network linking the SMA and putamen has been suggested as the neural locus for the storage of sequential motor memories, since lesions in these areas impair performance of well-learned sequences, but not the learning of new ones^12^. The current findings suggest that sequence generation in Condition 3 was predominantly under the control of automatic processes implemented in the network linking the SMA, putamen and anterior cerebellum. Furthermore, reward score-modulated activity was not observed in the striatum or in other cortical areas during the feedback period in Condition 3. Since predictions are nearly accurate in Condition 3, as demonstrated by the high reward score accuracy, subjects rarely encountered unexpected outcomes, which may explain the absence of a reward-modulated signal during the feedback period. These behavioral and neuroimaging results support the hypothesis that a motor-memory strategy takes control of well-learned action sequences. Conversely, activity in the left DLPFC, left ventrolateral premotor cortex, posterior parietal cortex, and anterior striatum were still observed after the delay period in both Conditions 1 and 2 (Fig. 7a,b) and suggests that action selection in these conditions was still under deliberative control during the execution time.

Neuroimaging studies with humans have also investigated the neural basis of sequential behavior using a variety of behavioral paradigms, such as the serial reaction time task^50^, the 2 × 10 sequence task^12^,^51^,^52^,^53^, and trial-and-error auditory feedback tasks^21^,^22^. One significant difference between previous experiments and the current study is that the grid-sailing task with pre-start delay explicitly tested that the use of internal models, explicitly represented in the KMs, for action sequence generation, while previous studies required sequential learning based on simple associations between finger movements and visual or auditory cues.

Recent studies of action learning and decision-making focused on the dichotomy between model-based and model-free action selection RL strategies, respectively^7^,^8^,^9^. In the current study, behavioral and neuroimaging results suggest a three-system action selection framework: an exploratory strategy playing a predominant role in early stages of learning, under the control of the network linking the VMOFC and anterior striatum; a model-based strategy takes control when a model of the environment is available and it is implemented in the network linking the DLPFC, dorsomedial striatum, and posterior cerebellum; and after extensive experience and acquisition of stereotypical movement patterns, a motor-memory, model-free strategy governs action selection via a network linking the proper-SMA, putamen, and anterior cerebellum. It is possible that particular types and orders of strategies used for action selection may depend on different features of the task, such as the steps of actions and the stochasticity of state transition, and the prior experience of the subject, as demonstrated in the current study. Despite the strong indication based on behavior and neuroimaging findings that subjects utilized different selection strategies, a limitation of the current study is the lack of use of computational models to substantiate our hypothesis and interpretations, and to assure that different action selection strategies were indeed used under each task condition. Future computational model-based studies may provide more compelling evidence for the current findings.

Perhaps the most important contribution of the present study is that it provides evidence that the network linking the prefrontal cortex, the posterior lateral cerebellum, and the dorsomedial striatum is likely to be involved in model-based action planning, or mental simulation. It remains unclear how models are learned, where action memories are stored in the brain, and how and at which levels (systems, molecular) the brain arbitrates between decision strategies. Switching from deliberative to automatic control may also depend on prefrontal-subcortical systems^54^. A recent model-based fMRI study with humans found evidence implicating the inferior lateral prefrontal cortex and frontopolar cortex in arbitration from model-free to model-based strategies for decision-making^43^. The neuromodulator, serotonin, has been suggested to regulate the time scale of future reward prediction^55^,^56^,^57^, but it may also regulate the process of model-based planning^58^. Additional studies will be needed to clarify exactly what function each of these brain areas performs, and how such functions are realized by local neural circuits.

## Methods

### Subjects

Eighteen healthy, right hand-dominant subjects (4 female; age 21–39, 26 ± 5 yrs), non-musicians, with normal vision, or vision corrected to normal (by contact lenses), participated in this study. All subjects were screened for history of psychiatric, neurological problems, or drug use at the time of the experiment. Subjects gave written informed consent and the experiment was approved by the ethics and safety committee of the Advanced Telecommunications Research Institute International (ATR), Kyoto, Japan, and they meet the Declaration of Helsinki requirements. Methods were carried out in accordance with approved guidelines. A fixed payment was made to subjects for their participation in this study. Due to technical fMRI pre-processing problems, we report the imaging results of only sixteen subjects.

### Apparatus

In the training session, performed outside the fMRI scanner, subjects sat comfortably at a table approximately 40–50 cm from the computer monitor. The task was run on a regular desktop computer and keyboard. Subjects used the index, middle, and ring fingers of the right hand to press the ‘v’, ‘b’, and ‘n’ keyboard buttons, respectively. Stimulus generation and behavioral data recording were done by custom-made programs written in MATLAB (version 7.0), using Psychophysics Toolbox functions (PTB-3). The test session was performed inside the fMRI scanner and the task was displayed through an imaging projector on a mirror inside the scanner mounted on top of the head coil. An fMRI-compatible response optic button box was used, obeying the same finger-key association used in the training session.

### Task Design

The goal of the “grid-sailing task” was to perform the shortest sequence of finger movements to move a cursor from its start position to a target goal position (Fermin *et al*., 2010). Subjects were given verbal instructions about task rules before training began, and received brief verbal feedback during the first training trials to ensure that they fully understood how to execute the task. The task started with an inter-trial interval of 3~5 s in which the computer displayed a 5 × 5 grid (10 cm × 10 cm) with a red fixation cross (FC) on top of the center square matching the monitor center position (Fig. 1). A trial started with presentation of a pair of start-goal (SG) positions together with a cursor (black triangle) on the start position. The shape of the cursor indicated which of the three KMs would be used during that trial. The color of the start position specified the start time condition to initiate a response: immediate start (green) – subjects were instructed to begin immediately after presentation of the SG and cursor; delayed start (red) – subjects had to wait 4~6 s for the go signal, when the start position switched from red to green. The color of the goal position was always blue. After the go signal, subjects were allowed a maximum response time of 6 s, which they were not explicitly informed of. During the response period, subjects performed key presses to move the cursor from the start position to the goal position. Immediate visual feedback of cursor position transition was provided after a keystroke. Any key press leading the cursor to leave the grid boundaries was invalid and was indicated by rapid blinking of the cursor, which did not move. A trial had three possible endings depending on the subject’s performance: a) successful trial with the execution of an optimal action sequence (shortest path to goal); b) successful trial with the execution of a suboptimal action sequence (longer path); c) unsuccessful trial due to failure to reach a goal position within the response time. Performance feedback containing the number of keystrokes and the reward score was displayed for 2 s right after the subject reached a goal position or following the end of the response time in unsuccessful trials. The reward was 100 points for the execution of an optimal action sequence; a discount of 5 points for each excessive keystroke for a suboptimal sequence, and 0 points for unsuccessful trials.

Three cursors, all triangular, were used in this experiment. For each, the acute angle indicated a direction (90 °, 180 °, 270 °) and was assigned to one of three different key-mappings (KM), which were associated with a three-move direction rule (Fig. 1a). Five different pairs of SG positions were prepared in advance (Fig. 1b). We created several combinations of KM and SG positions, so that specific KM-SG pairs could be used exclusively in training and test sessions (Fig. 1c). Initially, subjects knew neither KMs nor action sequences, and had to learn by trial-and-error. A target goal position could be reached via multiple optimal action sequences. However, in order to measure learning-related behavioral changes, subjects were instructed to learn a single optimal action sequence for each KM-SG pair, and to perform it as quickly and accurately as possible. No constraints were imposed on a sequence to be learned, and subjects were free to learn the action sequence they felt most comfortable performing.

Subjects performed one training session and one test session on consecutive days, with one night of sleep between sessions. Training and test sessions were further divided into three and two trial blocks, respectively, with trial blocks separated by 5-min rest intervals. Subjects were randomly assigned to one of three groups and remained in that group during the two experimental sessions. During training, only two KMs and four SG positions were employed by each group (Fig. 1c). Each of the KMs was combined with two different SG positions so that each subject group trained on four different KM-SG sets. A KM-SG set was practiced for 60 trials in the training session (20 trials per training block: 10 immediate start and 10 delayed-start trials). Immediate-start and delayed-start trials were alternated in short blocks of four trials, and each KM-SG set appeared randomly once in a short block.

During a test session, subjects performed the task inside the fMRI scanner, under three task conditions (Fig. 1c): Condition 1 – new KM: a KM not used in the training session was introduced and was combined with three SG pairs. Subjects had to learn both the new KM and an action sequence for each new KM-SG set. Condition 2 – learned KMs: KMs learned in the training session were combined with different SG pairs, and subjects essentially had to retrieve the appropriate KM rule and use it for planning and learning of new action sequences. Condition 3 – learned KM-SG sets: two of the four KM-SG sets practiced in the training session were chosen for re-testing. A single KM-SG set was practiced for 20 trials (10 trials per trial block: 5 immediate-start and 5 delayed-start trials). In total, subjects performed nine different KM-SG sets during the test session: three KM-SG sets in task Condition 1, four KM-SG sets in task Condition 2, and two KM-SG sets in task Condition 3. As in the training session, the task was performed in blocks of nine randomly generated trials, with alternation of the start time every nine trials. These task conditions were intended to mimic distinct levels of experience in action selection that might depend on implementation of specific action selection methods.

Subjects performed a short version (6 immediate- and 6 delayed-start trials) of the training task right before the start of fMRI scanning in order to ensure that they still remembered the task procedures, and more importantly, the action sequences learned for each KM-SG set on the previous day. At the end of this short pre-fMRI practice, subjects received explicit instruction regarding introduction of different task conditions that they had to perform inside the fMRI scanner.

### Behavior Analysis

We analyzed the following behavioral performance variables: a) reward score – number of points earned in a trial; b) over-step – number of excessive keystrokes executed to reach the target goal; c) reaction time (RT) – time elapsed from go signal to onset of the first keystroke; d) execution time (ET) – time from onset of the first keystroke to the execution of the last. These behavioral measures were separately subjected to a series of n-way ANOVA with Group (3), Task Condition (in the fMRI test session: Condition 1, Condition 2, and Condition 3), Trial Block (3 in the training session, and 2 in the fMRI test session), and Start Time Condition (immediate and delay) as factors. The statistical analysis also included multiple comparison procedures with Scheffe’s post-hoc test to seek for significant differences between pairs of levels.

### fMRI Data Acquisition

Scanning took place at the ATR Brain Activity Imaging Center, Kyoto, Japan. Detailed anatomical data were collected using a multiplanar, rapidly acquired gradient echo (MP-RAGE) sequence. T2*-weighted echo planar images (EPIs) with blood oxygen level-dependent (BOLD) contrast were acquired on a Siemens Tesla 3 Magnet Trio MRI scanner (TR: 2000 ms, TE: 23 ms, FOV: 192 mm, flip angle: 80 °). Thirty coronal oblique slices (3 × 3 × 5 mm) were acquired parallel to the plane containing the anterior and posterior commissures (AC-PC plane) after prescribing slice position based on automatic measurements of rotation, translation, and tilt of the structural images relative to an average image. The fMRI session was split into two runs, each lasting approximately 22 min, with the number of acquired imaging volumes dependent upon subject performance.

### Imaging Analysis

Statistical parametric mapping with SPM8 software (Wellcome Trust Centre for Neuroimaging, UCL) was used to preprocess all fMRI data, which included correction for slice time acquisition, spatial realignment of all volumes to the first image, spatial normalization using regressor parameters estimated by the unified segmentation process with voxel size resampled to 3 × 3 × 3 mm, and smoothed using a Gaussian kernel with an isotropic full width at half maximum of 8 mm. High-pass temporal filtering with a cutoff of 128 s was applied to remove low-frequency drifts in signal, and global changes were removed by proportional scaling.

Statistical analysis was conducted using a general linear model and a set of boxcar functions. Subject-specific design matrices were created, including two-trial events (Response Period and Reward delivery) of immediate-start condition trials and three events (Delay, Response Period and Reward delivery) of the delayed-start condition trials modulated separately by subject performance variables (number of keystrokes, execution time, and reward) for each of the three task conditions. A nuisance partition containing head motion regression parameters estimated in the realignment procedure was also entered in the design matrix. A series of contrast images were generated for each subject using a subtraction approach and were subsequently taken to a second-level for a random effects group analysis using one-sample t-tests. We were primarily interested in the specific effect of task condition on the delay-related period activity. Region of interest analysis (ROI) was carried out using the rfxplot toolbox (http://rfxplot.sourceforge.net/) and neuro-anatomically defined mask images generated using the WFU Pickatlas toolbox for the caudate and putamen, and the Anatomy Toolbox for the cerebellum. The Multicolor toolbox was used to plot voxels extracted from the basal ganglia and the cerebellum.

## Additional Information

**How to cite this article**: Fermin, A. S. R. *et al*. Model-based action planning involves cortico-cerebellar and basal ganglia networks. *Sci. Rep.* **6**, 31378; doi: 10.1038/srep31378 (2016).

## Supplementary Material

Supplementary Information
