# Supplementary material for: Model-based action planning involves cortico-cerebellar and basal ganglia networks
Source: Sci Rep. 2016 Aug 19;6:31378. doi: 10.1038/srep31378 (PMC4990901; doi:10.1038/srep31378)
Supplement: Supplementary Information [file srep31378-s1.pdf]

Supplementary Information

Model-based action planning involves cortico-cerebellar and basal ganglia networks

Alan S. R. Fermin,[1*] Takehiko Yoshida,[2] Junichiro Yoshimoto,[3] Makoto Ito,[4] Saori Tanaka,[5] Kenji Doya[4,*]

[1] Brain Science Institute, Tamagawa University, Tokyo 194-8610, Japan

[2] TBWA\HAKUHODO, Tokyo 105-0023, Japan

[3] Mathematical Informatics Laboratory, Nara Institute of Science and Technology, Nara 630-0192, Japan

[4] Neural Computation Unit, Okinawa Institute of Science and Technology, Okinawa 904-0495, Japan

[5] ATR Brain Information Communication Research Lab, Kyoto 619-0288, Japan

*Correspondence: ferminalan@gmail.com (A.S.R.F), doya@oist.jp (K.D.)

Supplementary Results

Analysis of Behavioral Data in the Training Session

REWARD SCORE

The reward score of KM-SG sets to be retested in the fMRI session as Condition 3 (Supplementary Figure 2A) was analyzed with a two-way ANOVA having as factors start time condition and trial block. This analysis revealed a significant effect of start time, $F(1,2154) = 31.21$, $p < 0.00001$, a significant effect of trial block, $F(2,2154) = 206.39$, $p < 0.00001$, and a significant interaction effect between start time condition and trial block, $F(2,2154) = 5.72$, $p = 0.003$. Sheffe's post hoc test showed that the mean reward score with a delayed start (M = 80.72, SE = 1.1088) was significantly higher than the reward score with an immediate start (M = 72.1, SE = 1.1088), $p < 0.0001$, and that reward score in trial block 3 (M = 92.2986, SE = 1.3365) was significantly greater than the reward score in trial block 2 (M = 81.8708, SE = 1.3365) and trial block 1 (M = 92.29886, SE = 1.3365), $p < 0.00001$. Supplementary Figure 2B shows the percentage of successful goal achievement, which includes suboptimal and optimal trials, and Supplementary Figure 2C shows the percentage of optimal goal attainment trials.

OVER-STEP

We estimated the number of excessive keystrokes that subjects executed to reach the goal (Supplementary Figure 2D). Two-way ANOVA showed a significant effect of start time, $F(1,2154) = 5.92$, $p < 0.0151$, and of trial block, $F(1,2154) = 139.88$, $p < 0.00001$, but failed to find any significance interaction between start time and trial block, $F(1,2154) = 0.24$, $p = 0.7858$. Scheffe's post hoc test showed that subjects were less efficient with an immediate start (M = 1.3043, SE = 0.0602) than with a delayed start (M = 1. 0972, SE = 0.0601), $p < 0.05$, and that the mean number of excess keystrokes was

larger in trial block 1 (M = 2.1842, SE = 0.0738) than in trial block 2 (M = 0.8959, SE = 0.0737; Block 3: M = 0.5222, SE = 0.0737).

REACTION TIME

The analysis of the reaction time (Supplementary Figure 2E) using a two-way ANOVA showed a significant effect of start time condition, $F(1,2154) = 1320.86$, $p < 0.00001$ and trial block, $F(2,2154) = 5.52$, $p = 0.0041$. The ANOVA failed to find an interaction between the start time and trial block, $F(2,2154) = 1.68$, $p > 0.\ 05$. The Scheffe’s post hoc test revealed that the mean reaction time with a delayed start (M = 0.560s, SE = 0.0179) was faster than the reaction time with an immediate start (M = 1.47s, SE = 0.0179), $p < 0.00001$, and reaction time decreased significantly across trial blocks with the reaction time in block 1 longer than (M = 1.06, SE = 0.0219) in block 3 (M = 0.9252, SE = 0.0219), $p < 0.05$.

EXECUTION TIME

Trial block had a significant effect on execution time (Supplementary Figure 2F), $F(2,2153) = 439.96$, $p < 0.00001$, but start time did not, $F(1,2153) = 1.26$, $p = 0.2617$. No interaction was found between start time and trial block, $F(2,2154) = 0.39$, $p = 0.6738$. Scheffe’s post hoc test revealed a significant decrease in execution time across trial blocks with a longer execution time in trial block 1 (M = 3.66, SE = 0.04) than in trial block 2 (M = 2.29, SE = 0.04) and trial block (M = 1.72, SE = 0.04).

Analysis of Behavioral Data in the Test Session

REWARD SCORE

We merged data of subjects in all three groups and carried out the statistical analysis with the null hypothesis of no significant difference in the mean reward score among the three task conditions. A four-way ANOVA found no effect of group condition, F(2,3204) = 2.74, p = 0.0649. The analysis found a significant effect of task condition, F(2,3204) = 186.54, p < 0.0001, start time condition, F(1,3204) = 131.64, p < 0.0001, and trial block, F(1,3204) = 126.09, p < 0.0001. No two-way interaction effects were found between group and test conditions, F(4,3204) = 1.95, p = 0.0999, or between group and start conditions, F(2,3204) = 0.54, p = 0.5803. Significant two-way interactions were found between group and trial block conditions, F(2,3204) = 5.7, p = 0.0034, test and start time conditions, F(2,3204) = 20.13, p < 0.0001, test and trial block conditions, F(2,3204) = 25.96, p < 0.0001, and start time and trial block conditions, F(1,3204) = 12.55, p = 0.0004. No three-way interaction effects were found for group x test x start time conditions, F(4,3204) = 0.25, p = 0.9124, for group x test x trial block, F(4,3204) = 1.7, p = 0.1467, or for group x start x trial block conditions, F(2,3204) = 0.06, p = 0.9397. Significant three-way interaction effects were found for test x start time x trial block conditions, F(2,3204) = 4.33, p = 0.0133. No four way interaction effects were found, F(4,3204) = 1.21, p < 0.3037).

Scheffe’s post hoc test (p < 0.00001) showed that the overall reward score in Condition 1 (M = 64.03, SE = 1.08) was significantly smaller than that of Condition 2 (M = 72.77, SE = 0.94), and that the reward score of Condition 3 (M = 96.77, SE = 1.33) was the largest. Scheffe’s post hoc test showed no significant increase in the reward score of Condition 3 across trial blocks (block 1: M = 96.70, SE = 1.88; block 2: M = 96.84, SE = 1.88, p > 0.05), whereas a significant increase was observed in Condition 1 (block 1: M = 52.84, SE = 1.54; block 2: M = 75.22, SE = 1.54, p < 0.00001) and Condition 2 (block 1: M = 61.97, SE = 1.33; block 2: M = 83.56, SE = 1.33, p < 0.00001). The post hoc test also

showed that the average reward score in Condition 2 was significantly higher than in Condition 1 in trial blocks 1 ($p < 0.0013$) and 2 ($p < 0.005$).

We investigated in detail the effect of immediate and delayed starts on the ability of subjects to plan and execute successful action sequences. Scheffe’s post hoc test showed a significant effect of start time on the reward score of Condition 1 (immediate: $M = 55.52$, $SE = 1.54$; delay: $M = 72.53$, $SE = 1.54$, $p < 0.00001$), Condition 2 (immediate: $M = 60.56$, $SE = 1.33$; delay: $M = 84.97$, $SE = 1.33$, $p < 0.00001$), but no significant difference in Condition 3 (immediate: $M = 94.94$, $SE = 1.88$; delay: $M = 98.61$, $SE = 1.88$, $p = 0.8645$). Post hoc analysis revealed that the reward score in Condition 2 under a delayed start was significantly larger in relation to Condition 1, $p < 0.001$, but no difference was found between the two conditions for performances with an immediate start, $p > 0.05$.

The benefit of the delayed start on reward score was further investigated by calculating the reward gain, defined as the difference between the reward score of the delayed- and immediate-start trials for each KM-SG set. The reward gain was first computed individually and then taken to a group analysis and tested with a two-way ANOVA with task condition and trial block as factors. This analysis showed a significant effect of task condition, $F(2,1614) = 25.53$, $p < 0.00001$, trial block, $F(1,1614) = 15.92$, $p < 0.0001$ and a two-factor interaction, $F(2,1614) = 5.49$, $p = 0.0042$. A post-hoc analysis also revealed that the overall reward gain was larger in Condition 2 ($M = 24.40$, $SE = 1.67$, $p = 0.0118$) than in Condition 1 ($M = 17.01$, $SE = 1.93$) and the reward gain in Condition 3 ($M = 3.66$, $SE = 2.37$) was the smallest (comparison with condition 1: $p < 0.00001$; comparison with condition 2: $p < 0.00001$). The post hoc analysis also showed that the difference in reward gain was significant between Conditions 1 and 2 in block 1 ($p = 0.0162$), but not in block 2 ($p = 0.9662$). While no significant decrease was observed in the

reward gain between trial blocks for Condition 1 ($p = 0.1262$), a significant decrease was observed in Condition 2 ($p < 0.00001$).

The beneficial effect of the delay was also estimated by calculating the improvement in successful trials (suboptimal + optimal trials) and in optimal trials (reward score = 100). Both measures were estimated similarly to the estimation of the reward score improvement. For both measures the gain was first computed individually and then taken to a group analysis and tested with a two-way ANOVA with task condition and trial block as factors. For the improvement in successful trials, this analysis found no significant effect of trial block, $F(1,1614) = 2.43$, $p = 0.1191$, or for the interaction between task condition and trial block, $F(2,1614) = 2.54$, $p < 0.0795$. A significant effect was found for task condition, $F(2,1614) = 110.85$, $p < 0.00001$. Scheffe’s post hoc test found a significant difference ($p < 0.00001$) in improvement between Condition 1 ($M = 0.2183$, $SE = 0.0129$) and Condition 2 ($M = 0.3196$, $SE = 0.0112$), and with Condition 3 ($M = 0.0316$, $SE = 0.0158$) having the least improvement compared with Condition 1 ($p < 0.00001$) and Condition 2 ($p < 0.00001$). The improvement in Condition 2 (block 1: $M = 0.3537$, $SE = 0.0158$; block 2: $M = 0.2854$, $SE = 0.0158$) was significantly larger than the improvement in Condition 1 (block 1: $M = 0.2278$, $SE = 0.0182$; block 2: $M = 0.2089$, $SE = 0.0182$) in both trial blocks (block 1: $p < 0.00001$; block 2: $p = 0.0232$). While no significant difference was found in the improvement of Condition 1 between trial blocks ($p = 1$), a significant difference was found in Condition 2 ($p = 0.034$). No significant difference in improvement was found in Condition 3 between trial blocks (block 1: $M = 0.0243$, $SE = 0.0223$; block 2: $M = 0.0389$, $SE = 0.0223$; $p = 1$).

The two-way ANOVA of the improvement in optimal trials found a significant effect of task condition, $F(2,1614) = 64.85$, $p = 0.00001$, but no significant effect of trial block, $F(1,1614) = 0.07$, $p = 0.7844$,

and no significant interaction, $F(2,1614) = 0.14$, $p = 0.8696$. Scheffe’s post hoc analysis found that the improvement in optimal trials was significantly larger in Condition 2 (M = 0.2706, SE = 0.0114) than in Condition 1 (M = 0.1297, SE = 0.0131), $p < 0.00001$, and that Condition 3 (M = 0.0647, SE = 0.0161) had the least improvement (comparison with Condition 1: $p = 0.0075$; comparison with Condition 2: $p < 0.00001$). The post hoc analysis also found that the improvement in Condition 2 (block 1: M = 0.2713, SE = 0.0161; block 2: M = 0.2699, SE = 0.0161) was significantly larger than in Condition 1 (block 1: M = 0.1272, SE = 0.0186; block 2: M = 0.1323, SE = 0.0186) in both trial blocks (block 1: $p < 0.00001$; block 2: $p < 0.00001$).

Next, we classified the trials into three types based on their reward score (error trials, reward score = 0, suboptimal trials, 0 < reward score < 100, and optimal trials, reward score = 100), and performed a three-way ANOVA with task condition, start time condition and trial type as main factors. ANOVA demonstrated a significant effect for trial type, $F(2,3204) = 120.36$, $p < 0.00001$, interaction effect for task condition and trial type, $F(4,3204) = 205.97$, $p < 0.00001$, start time condition and trial type, $F(2,3204) = 97.15$, $p < 0.00001$, and three way interaction, $F(4,3204) = 15.85$, $p < 0.00001$. Scheffe’s post hoc test showed that the overall error rate in Condition 1 (M = 0.32, SE = 0.0125) was significantly higher than in Condition 2 (M = 0.23, SE = 0.0108, $p = 0.0006$), and Condition 3 had the lowest error rate (M = 0.02, SE = 0.0153; comparison with Condition 1: $p < 0.00001$; comparison with Condition 2: $p < 0.00001$). Furthermore, the post hoc test also showed the rate of optimal trials in Condition 2 (M = 0.54, SE = 0.0108) was significantly higher than in Condition 1 (M = 0.46, SE = 0.0125), $p = 0.0053$. The latter effect was also significant ($p = 0.0069$) in delayed-start trials between Condition 2 (M = 0.65, SE = 0.0153) and Condition 1 (M = 0.52, SE = 0.0177).

Since there were three groups of subjects performing the same task conditions, we further conducted a post hoc analysis to seek for significant differences between subject groups during the same task condition (Supplementary Figure). Scheffe's post hoc test did not find any significant difference between groups regardless of condition (Condition 1: group 1 x group 2: $p = 0.4267$; group 1 x group 3: $p = 0.4296$; group 2 x group 3: $p = 1$; Condition 2: group 1 x group 2: $p = 1$; group 1 x group 3: $p = 0.8896$; group 2 x group 3: $p = 0.9364$; Condition 3: group 1 x group 2: $p = 0.9999$; group 1 x group 3: $p = 1$; group 2 x group 3: $p = 1$).

The mean absolute deviation (MAD) was calculated on a trial-by-trial basis as a measure of variability of performance with reward score as the variable of interest. MAD was estimated for each subject separately for each KM-SG set and start time condition and then a group analysis was performed using three-way ANOVA, with task condition, start time condition and trial block as factors. This analysis revealed a significant effect of task condition, $F(2,636) = 82.95$, $p < 0.00001$, start time, $F(1,636) = 27.52$, $p < 0.00001$, trial block, $F(1,636) =$, $p < 0.00001$, and a two-way interaction effect for task condition and trial block, $F(2,636) = 8.09$, $p < 0.0003$. No significant interaction effects were found for task condition and start condition, $F(2,636) = 2.35$, $p = 0.0964$, start time and trial block, $F(1,636) = 0.64$, $p = 0.4248$ or for a three-way interaction, $F(2,636) = 0.15$, $p = 0.8573$. The post hoc test indicated that there was significantly higher variability in the reward score in Condition 1 ($M = 24.64$, $SE = 1.03$) than in Condition 2 ($M = 20.96$, $SE = 0.89$), $p = 0.0199$. Condition 3 ($M = 4.41$ $SE = 1.27$) had the lowest variability (comparison with Condition 1: $p < 0.00001$; comparison with Condition 2: $p < 0.00001$).

EXCESS STEPS

A three-way ANOVA found a main effect of task condition, $F(2,3228) = 109.92$ $p < 0.00001$, trial block, $F(1,3228)=19.85$, $p < 0.00001$, two-way interaction effect for task condition and start time, $F(1,3228) = 2.88$, $p < 0.05$, and task condition and trial block, $F(1,3228) = 6.19$, $p = 0.0021$. No other two-way or three-way interactions were found. Scheffe's post hoc test indicated that the average number of excess steps in Condition 1 ($M = 1.75$, $SE = 0.063$) was larger than in Condition 2 ($M = 1.43$, $SE = 0.054$), $p = 0.0006$ or Condition 3 ($M = 0.3$, $SE = 0.077$), $p < 0.00001$. Subjects in Condition 2 used more steps than in Condition 3 ($p < 0.00001$). The number of excess steps did not differ between Condition 1 ($M = 1.96$, $SE = 0.0892$) and Condition 2 ($M = 1.74$, $SE = 0.0773$) in trial block 1 ($p = 0.6256$), but was significantly different in trial block 2 ($p = 0.0264$) with the number of extra steps being larger in Condition 1 ($M = 1.53$, $SE = 0.0892$) than Condition 2 ($M = 1.11$, $SE = 0.0773$). The mean absolute deviation, as a measure of performance variability, was estimated separately for each subject, KM-SG set, and trial block, and was also analyzed in a three-way ANOVA with task condition, start time condition, and trial block as factors of interest. We found significant effect of test condition, $F(2,636) = 58.54$, $p < 0.00001$, effect of trial block, $F(1,636) = 6.59$, $p = 0.0105$, and interaction effect between task condition and trial block, $F(1,636) = 3.28$, $p = 0.0382$. Scheffe's post hoc analysis showed that subjects varied more in the number of extra steps employed in Condition 1 ($M = 1.36$, $SE = 0.0597$) than in Condition 2 ($M = 1.14$, $SE = 0.0517$), $p = 0.0233$, and Condition 3 ($M = 0.36$ $SE = 0.0731$), $p < 0.00001$. Excess step variability was also higher in Condition 2 than in Condition 3 ($p < 0.00001$). The comparison between trial blocks also showed no significant difference in extra step variability in Condition 1 (block 1: $M = 1.50$, $SE = 0.0845$; block 2: $M = 1.21$, $SE = 0.0845$; $p = 0.3501$). On the other hand, a significant decrease in extra step variability occurred in Condition 2 (block 1: $M = 1.32$, $SE = 0.0731$; block 2: $M = 0.96$, $SE = 0.0731$; $p = 0.0339$). No significant difference in excess step variability

was observed in Condition 3 between trial blocks (block 1: M = 0.3233, SE = 0.1034; block 2: M = 0.41, SE = 0.1034; p = 0.9959).

REACTION TIME

A three-way ANOVA on the reaction time with task condition, start time condition, and trial block as main factors found a main effect of task condition, $F(2,3228) = 72.85$, $p < 0.00001$, start time, $F(1,3228) = 2344.04$, $p < 0.00001$, and trial block, $F(1,3228) = 24.12$, $p < 0.00001$. Significant two-way interactions were found for task condition and start time, $F(1,3228) = 16.34$, $p < 0.00001$, and a nearly significant effect for task condition and trial block was observed $F(2,3228) = 2.8$, $p = 0.0611$. No interaction was found for start time and trial block, $F(2,3228) = 0.4$, $p = 0.5263$, or for three-way interaction, $F(2,3228) = 1.1$, $p = 0.3326$.

Scheffe’s post hoc test showed no significant difference in overall reaction time between Condition 1 (M = 1.34, SE = 0.0212) and Condition 2 (M = 1.38, SE = 0.0184), p = 0.7136. This analysis found that the average reaction time in Condition 3 (M = 1.01, SE = 0.0260) was significantly faster than in Conditions 1 and 2, $p < 0.00001$. Post hoc analysis also found a significantly slower reaction time in immediate- (M = 1.86, SE = 0.0180) than in delayed-start trials (M = 0.63, SE = 0.0180), $p < 0.00001$, and a significant decrease in reaction time between trial blocks (block 1: M = 1.31, SE = 0.0180; block 2: M = 1.18, SE = 0.0180), $p < 0.0001$. In immediate-start trials, the reaction time in Condition 3 (M = 1.5197, SE = 0.0368) was significantly faster than the reaction time in Condition 1 (M = 2.03, SE = 0.03; $p < 0.00001$) and Condition 2 (M = 2.0504, SE = 0.0260; $p < 0.00001$). Similarly, the reaction time in Condition 3 (M = 0.5099, SE = 0.0368) in delayed-start trials was significantly faster than the reaction time in Condition 1 (M = 0.6698, SE = 0.03; $p < 0.0451$) and Condition 2 (M = 0.7157, SE = 0.0260; p

< 0.00001). The reaction time in Condition 1 and Condition 2 did not differ between immediate- (p = 0.9983) and delayed-start trials (p = 0.9311). No significant difference in reaction time was observed in Condition 1 between the immediate-start trial blocks 1 (M = 2.1110, SE = 0.0424) and 2 (M = 1.9490, SE = 0.0424), p = 0.2265, or between the delayed-start trial blocks 1 (M = 0.7429, SE = 0.0424) and 2 (M = 0.5967, SE = 0.0424), p = 0.8776. Similarly, no significant difference in reaction time was observed in Condition 2 between the immediate-start trial blocks 1 (M = 2.1105, SE = 0.0368) and 2 (M = 1.9902, SE = 0.0368), p = 0.4661. No significant difference in reaction time was found between Condition 1 and 2 in trial block 1 (p = 1) and in trial block 2 (p = 0.9999). On the other hand, a significant difference in reaction time was observed in Condition 2 between the delayed-start trial blocks 1 (M = 0.8409, SE = 0.0368) and 2 (M = 0.5906, SE = 0.0368), p = 0.0169.

EXECUTION TIME

The three-way ANOVA with task conditions, start time condition and trial block as main factors found a significant main effect of task condition, $F(2,3228) = 325.86$, $p<0.00001$, start time, $F(1,3228) = 4.66$, $p = 0.0309$, and trial block, $F(1,3228) = 170.16$, $p<0.00001$, and a two-way interaction between task condition and trial block, $F(1,3228) = 19.06$, $p<0.00001$. No significant effects were found for other interactions. Scheffe's post hoc test showed the average execution time in Condition 1 (M = 3.173, SE = 0.0349) was significantly longer than in Condition 2 (M = 2.9211, SE 0.0302), p < 0.00001 and in Condition 3 (M = 1.820, SE = 0.0427), p < 0.00001. Execution time in Condition 2 was also significantly longer than in Condition 3 (p < 0.00001). During immediate-start trials, execution times in Condition 1 (M = 3.0650, SE = 0.0494) and Condition 2 (M = 2.9044, SE = 0.0427) did not differ significantly (p = 0.3020). However, a significant difference in execution time was observed between Condition 1 (M = 3.2826, SE = 0.0494) and Condition 2 (M = 2.9377, SE = 0.0427) with a delayed start

($p < 0.00001$). Execution time decreased significantly in Condition 1 between immediate start-trial blocks (block 1: M = 3.3538, SE = 0.0698; block 2: M = 2.7761, SE = 0.0698; $p < 0.00001$) and delayed-start trial blocks (block 1: M = 3.7106, SE = 0.0698; block 2: M = 2.8545, SE = 0.0698; $p < 0.00001$). A significant decrease in execution time across trial blocks was also observed in Condition 1 between immediate-start trial blocks (block 1: M = 3.288, SE = 0.0605; block 2: M = 2.55, SE = 0.0605; $p < 0.00001$) and delayed-start trial blocks (block 1: M = 3.3554, SE = 0.0605; block 2: M = 2.5200, SE = 0.0605; $p < 0.002$). No significant differences in the execution time were observed in Condition 3 across immediate- (block 1: M = 1.8827, SE = 0.0855; block 2: M = 1.7377, SE = 0.0855; $p = 0.9997$) or delayed-start trial blocks (block 1: M = 1.9113, SE = 0.0855; block 2: M = 1.7510, SE = 0.0855; $p = 0.9992$).

A MAD analysis using the same procedures as described in the analysis of reward score was carried out to examine performance variability in execution time. Three-way ANOVA with repeated measures revealed a significant effect of task condition, $F(2,48)=112.54$, $p < 0.001$, start time, $F(1,48)=37.89$, $p < 0.001$, trial block, $F(1,48)=10.62$, $p < 0.005$, and significant two-way interaction for task condition and start time, $F(2,48)=6.64$, $p < 0.005$, and test condition and trial block, $F(2,48)=8.95$, $p < 0.001$. Post hoc analysis showed higher variability in execution time in Condition 1 (M = 1065, SE = 47) than in Condition 2 (M = 945, SE = 28) and Condition 3 (M = 553, SE = 30).

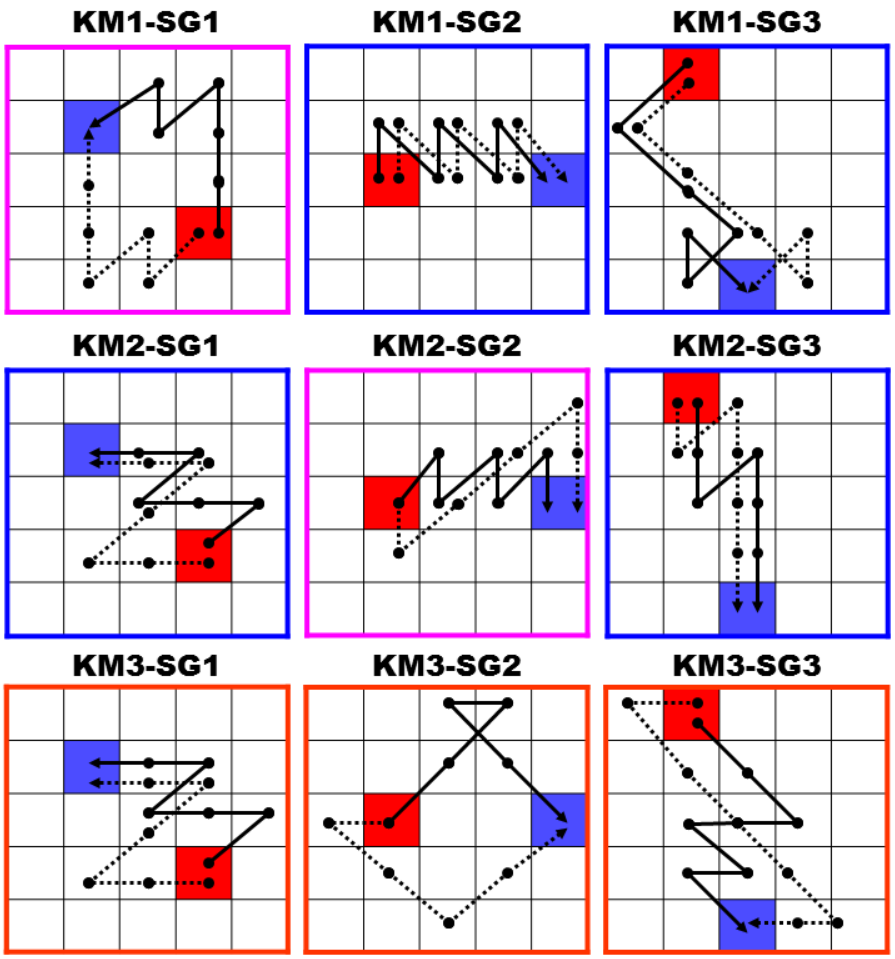

Supplementary Figure S1: Examples of optimal paths performed for the 3x3 combinations of KMs and SGs in the test session by two representative subjects (dotted lines: subject 02; continuous line: subject 16). The color coded grids represent the task conditions to which each KM-SG combination belonged: Condition 1 (orange), Condition 2 (blue) and Condition 3 (pink).

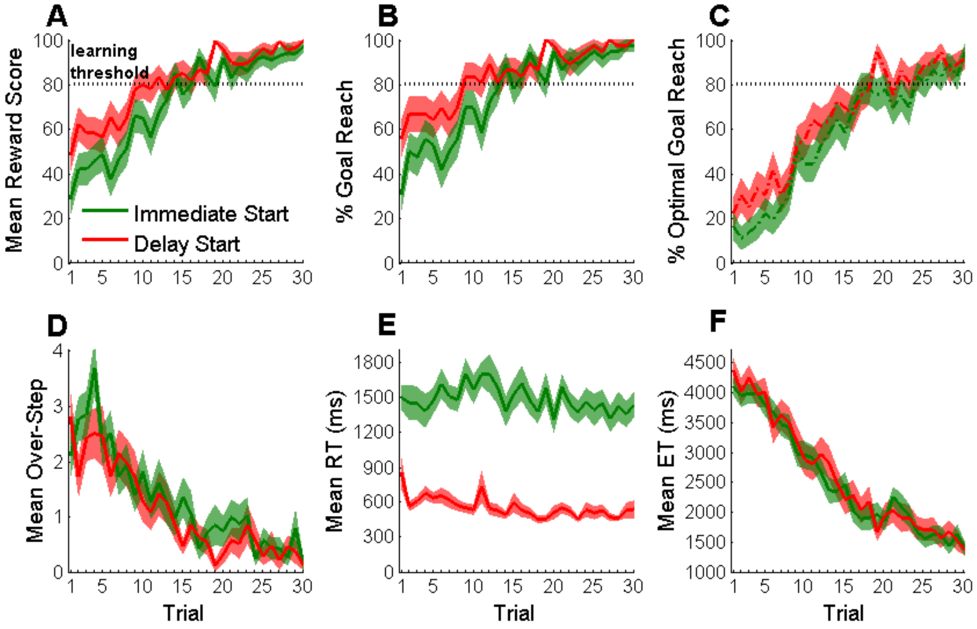

Supplementary Figure S2: Behavioral performance in the training session. A: average reward score. B: average percentage of trials in which subjects successfully reached the goal position. C: average percentage of trials in which subjects reached the goal by performing optimal (shortest path) action sequences. D: average number of steps performed above the optimal sequence size to reach the goal position. E: average reaction time (RT). F: average execution time (ET). The ET was measured as the time between the first and last keystrokes in a successful action sequence. Behavior shown in D, E and F was analyzed only for successful trials.

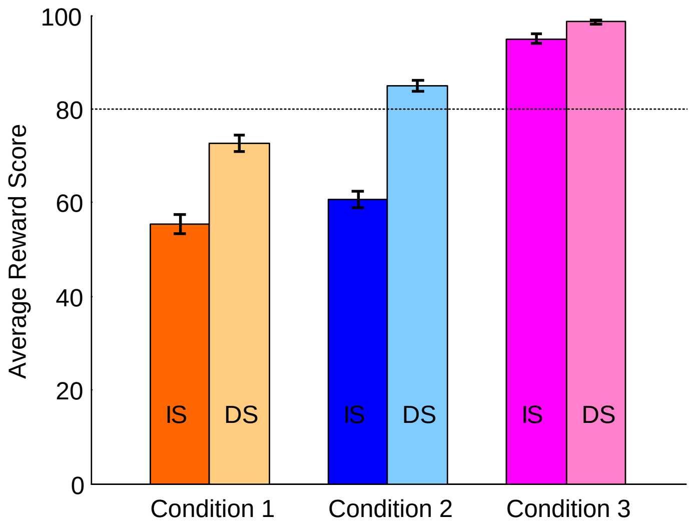

Supplementary Figure S3: Average reward score for each task condition for immediate-start (IS) and delayed-start (DS) trials. Condition 1: new KM; Condition 2: learned KM with new SG positions; Condition 3: learned KM-SG sets.

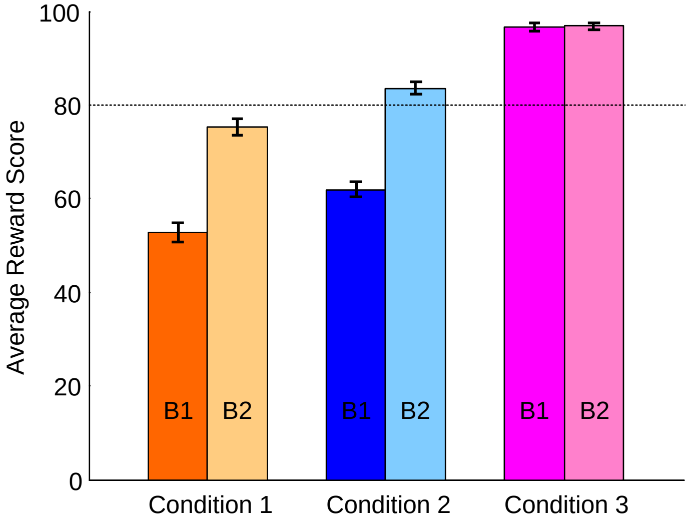

Supplementary Figure S4: Average reward score for each task condition separated by trial block (B1: trial block 1; B2: trial block 2), each block composed of 10 trials for each task condition. Condition 1: new KM; Condition 2: learned KM with new SG positions; Condition 3: learned KM-SG sets. While the reward score performance in Condition 3 was highly accurate and not significantly different between trial blocks ($p = 0.86$), the reward score significantly increased in trial block 2 for Conditions 1 ($p < 0.0001$) and 2 ($p < 0.0001$).

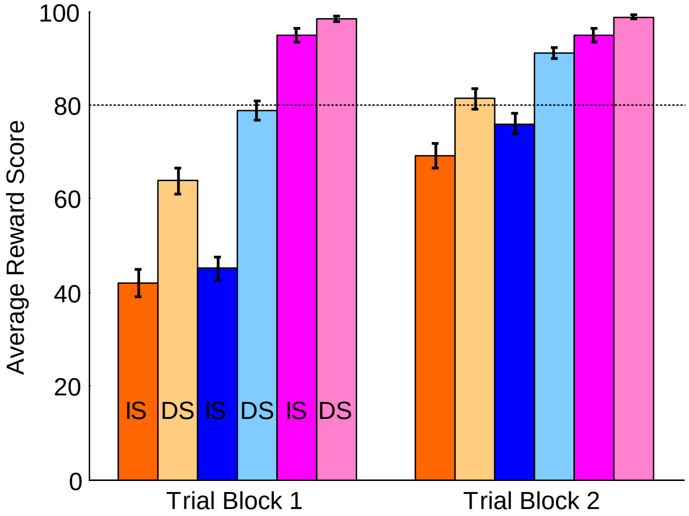

Supplementary Figure S5: Average reward score for each task condition, separated by trial block and immediate-start (IS) and delayed-start (DS) trials. The ANOVA analysis revealed a significant increase in the reward scores of Conditions 1 and 2 across trials blocks, indicating that subjects could successfully improve their performance as learning went on. Conversely, no significant differences in performance across trial blocks were observed in Condition 3. Condition 1 (dark orange and light orange bars): new KM; Condition 2 (dark blue and light blue bars): learned KM with new SG positions; Condition 3 (dark purple and light purple bars): learned KM-SG sets.

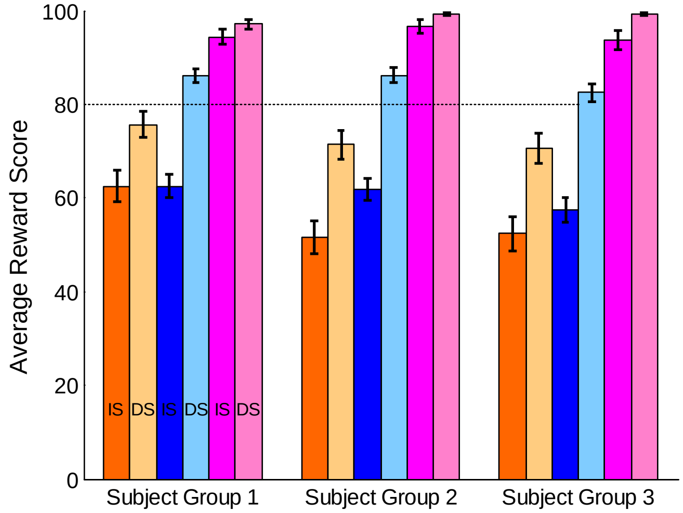

Supplementary Figure S6: Average reward score for each task condition, separated start time condition (immediate-start: IS; delayed-start: DS) and subject group. The ANOVA did not find significant differences in performance among the three subject groups, suggesting a comparable effect of task condition on reward score regardless of which KM-SG sets were assigned for the subjects under each task condition.

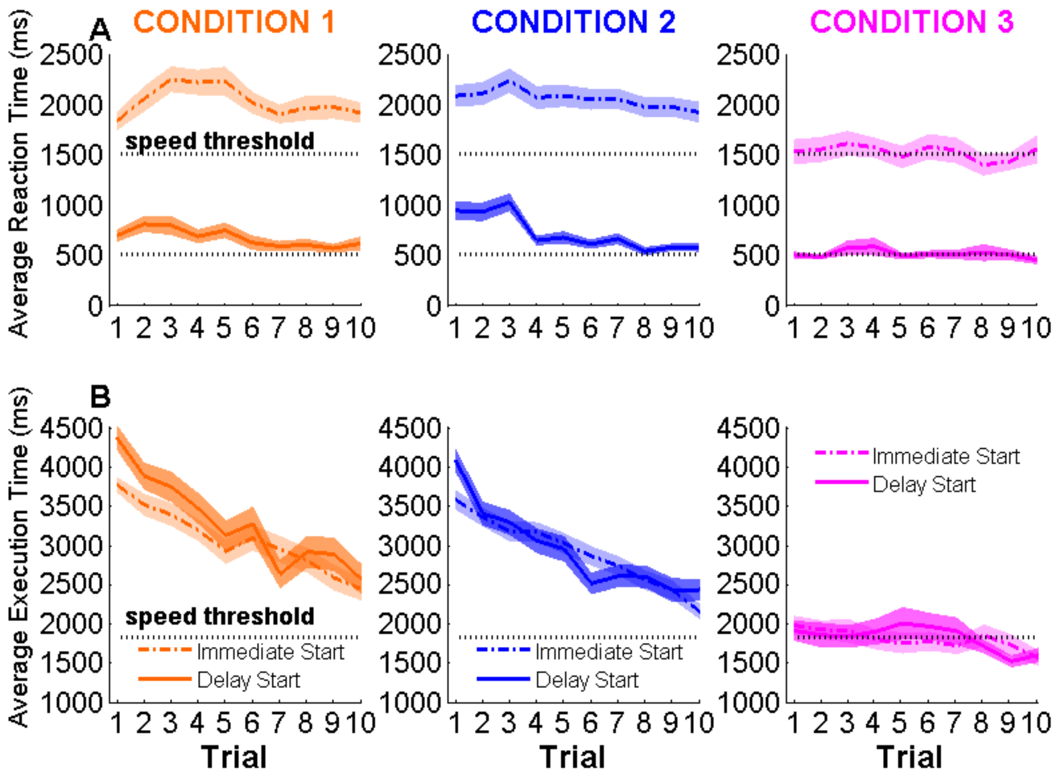

Supplementary Figure S7: Performance speed measures in the fMRI session, separated for each task condition (Condition 1: left orange panels; Condition 2: middle blue panels; Condition 3: right purple panels), and for immediate start (colored broken lines) and delayed-start (continuous lines) trials. **A (top row)**: Average reaction time (ms). The top and bottom black dotted lines represent the overall mean reaction time in Condition 3 in delayed-start and immediate-start trials, respectively. **B (bottom row)**: Average execution time (ms). The black dotted line represents the overall mean execution time in Condition 3.

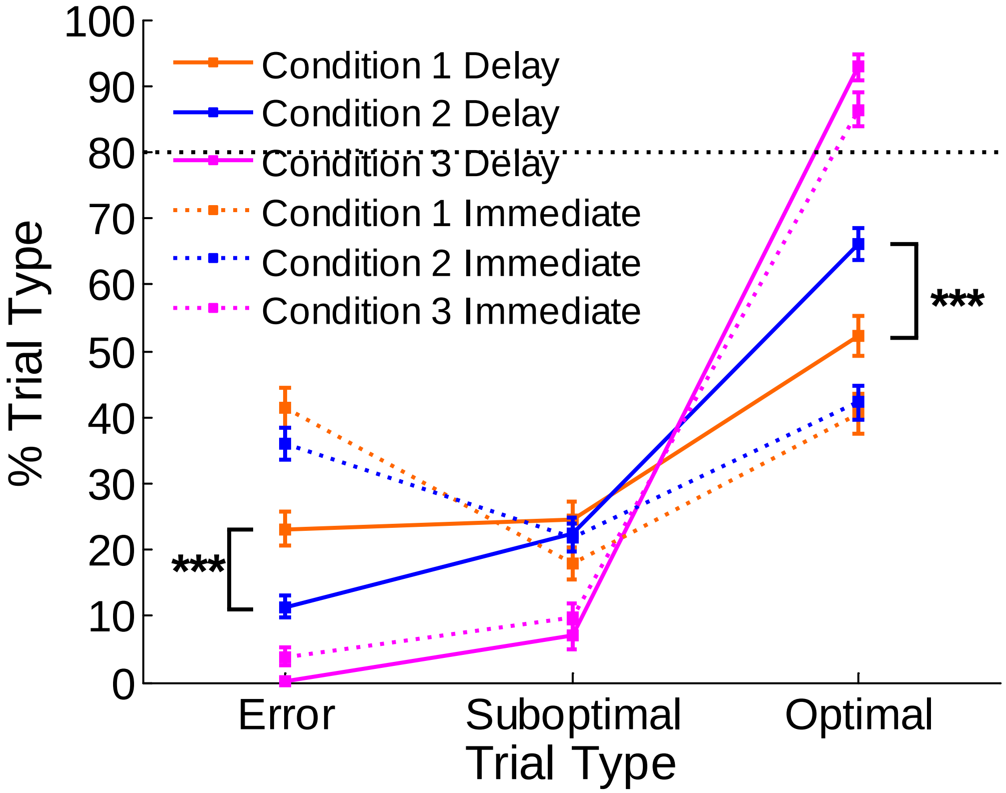

Supplementary Figure S8: Behavior performance in the fMRI test session separated by trial type. Trials were classified as error (reward score = 0), suboptimal (0 < reward score < 100) and optimal (reward score = 100) based on the reward scored subjects received at the end of a trial.

Table S1. Comparison of neural activity in conditions requiring learning (Condition 1 and Condition 2) versus automatic behavior (Condition 3). Related to results in Figure 4.

Brain region	Hemi	Coordinates [x,y,z]			T-value	P (FWE)	SVC (FWE)	P (unc.)
Subtraction Condition 1 > Condition 3 of results presented in Figure 4A, 4C, 4D								
DLPFC	L	-39	23	31	6.11	0.05		0.0001
Inferior parietal cortex	R	39	-49	52	6.08	0.0001		0.0001
Precuneus	R	12	-61	55	5.86		0.0001	0.0001
Supramarginal	R	54	-28	40	5.47		0.0001	0.0001
Anterior putamen	R	15	14	-2	5.91	0.003		0.0001
Anterior caudate nucleus	R	9	17	7	5.33		0.0001	0.0001
Medial globus pallidus	L	-12	-4	-5	5.79	0.4	0.004	0.0001
Superior frontal gyrus	R	24	5	52	5.79	0.0001		0.0001
Ventrolateral premotor cortex	R	45	11	19	5.77	0.005		0.0001
Dorsal premotor cortex	L	-18	11	43	5.73	0.001		0.0001
Middle frontal gyrus	L	-24	8	58	5.28		0.0001	0.0001
SMA	L	-3	20	49	4.61		0.002	0.0001
Anterior caudate nucleus	L	-12	14	1	5.7	0.028		0.0001
Inferior parietal cortex	L	-45	-49	46	5.47	0.001		0.0001
Precentral gyrus	L	-45	2	40	5.12	0.1	0.004	0.0001
Inferior temporal gyrus	L	-51	-55	-8	4.21	0.7	0.009	0.0001
Subtraction Condition 2 > Condition 3 of results presented in Figure 4B, 4C, 4D								
Ventrolateral premotor cortex	R	51	8	25	7.82	0.002		0.0001
Inferior parietal cortex	R	42	-43	46	7.15	0.0001		0.0001
Middle occipital gyrus	R	39	-67	25	6.20		0.001	0.0001
Supramarginal	R	54	-28	42	5.96		0.001	0.0001
DLPFC	L	-48	32	34	6.98	0.0001		0.0001
Precentral gyrus	L	-45	2	40	5.99		0.001	0.0001
Anterior caudate nucleus	L	-12	11	1	6.28	0.0001		0.0001
Anterior caudate nucleus	R	12	11	1	6.04		0.0001	0.0001
Superior frontal gyrus	R	24	8	55	6.02	0.0001		0.0001
Superior frontal gyrus	L	-21	8	61	6.01	0.0001		0.0001
SMA	L	-6	23	49	5.99		0.001	0.0001
Anterior cerebellum	L	-33	-46	-29	5.63	0.1	0.002	0.0001
Inferior temporal gyrus	L	-54	-58	-11	5.48	0.49	0.005	0.0001
DLPFC	R	45	38	19	5.27	0.1	0.001	0.0001
Inferior frontal gyrus	L	-36	44	1	5.02	0.6	0.007	0.0001
Inferior precuneus	R	15	-55	19	4.76	0.6	0.006	0.0001
Superior precuneus	L	-9	-58	58	5.06		0.0001	0.0001

P (FWE): statistical test whole-brain corrected for multiple comparison with threshold at the cluster level.

SVC (FWE): small volume correction (SVC) with sphere radius of 10 mm corrected for multiple comparisons with threshold at the cluster level.

P (unc.): statistical test whole-brain uncorrected.

Hemi: hemisphere – L (left), R (right).
-DLPFC: dorsolateral prefrontal cortex; SMA: supplementary motor area.

Table S2. Comparison of neural activity between Conditions 1 (value-based strategy) and Condition 2 (model-based strategy). Related to results in Figure 5.

Brain region	Hemi	Coordinates [x,y,z]			T-value	P (FWE)	SVC (FWE)	P (unc.)
Subtraction Condition 1 > Condition 2 of results presented in Figure 5A								
Anterior prefrontal cortex	R	9	56	-17	4.66	0.025		0.001
Ventromedial OFC	R	6	26	-17	4.56	0.015		0.001
Anterior caudate nucleus	R	12	20	4	3.17	0.023		0.001
Precuneus	L	-12	-43	52	4.51		0.016	0.001
Supramarginal	L	-42	-49	19	4.27	0.023		0.001
Supramarginal	R	51	-31	34	4.14		0.028	
Postcentral	L	-66	-13	25	4.10		0.030	0.001
Dorsal premotor cortex	L	-15	17	61	4.06		0.032	0.001
Dorsal premotor cortex	R	21	17	58	3.71		0.045	0.001
Cerebellum Crus 1	L	-27	-82	-29	3.71		0.045	0.001
Anterior caudate	L	-9	17	-8	4.63	0.009		0.001
DLPFC	R	39	35	46	4.61	0.01		0.001
Postcentral	R	54	-13	31	4.24		0.020	0.001
Middle temporal gyrus	R	51	-76	16	4.18		0.023	0.001
Subtraction Condition 2 > Condition 1 of results presented in Figure 5B								
Superior medial PFC	R	9	47	31	5.51	0.001		0.001
Superior medial PFC	L	-15	32	40	4.23	0.003		0.001
Inferior precuneus	L	-9	-55	10	4.63	0.001		0.001
Anterior insula	L	-36	20	-8	4.75		0.048	0.001
Inferior frontal gyrus	L	-42	23	1	3.14		0.025	0.001
Anterior cerebellum	L	-18	-43	-35	4.49	0.002		0.000
Parahippocampus	L	-21	-31	-17	3.96		0.05	0.001
Inferior frontal gyrus	R	36	26	16	4.19		0.03	0.001
Anterior cingulate	R	6	23	25	4.11	0.004		0.001
Middle cingulate	L	-9	17	31	3.27	0.008		0.001
DLPFC	L	-57	17	34	4.07	0.004		0.001
Lateral orbitofrontal cortex	L	-48	47	-2	3.93	0.005		0.001
Posterior cerebellum	L	-6	-52	-50	3.91	0.005		0.001
SMA	R	9	20	67	3.06	0.029		0.001
Middle temporal gyrus	R	60	-4	-23	3.67	0.009		0.001
Inferior frontal gyrus	L	-54	32	16	3.59	0.010		0.001
Dorsal caudate nucleus	L	-9	20	-5	4.39	0.015		0.001
Dorsal caudate nucleus	R	12	2	18	4.17	0.023		0.001
Superior parietal lobe	L	-33	-70	43	3.42	0.014		0.001
Subtraction Condition 3 > baseline of results presented in Figure 5C								
Precentral gyrus	L	-33	-22	61	11.41	0.0001		0.0001
Postcentral gyrus	L	-39	-34	49	9.92	0.0001		0.0001
Supramarginal	L	-57	-22	40	9.62	0.0001		0.0001
Anterior cerebellum	R	33	-55	-23	9.27	0.0001		0.0001
Superior parietal cortex	R	24	-64	61	7.91	0.0001		0.0001
Precuneus	R	18	-67	52	7.11	0.0001		0.0001

Lateral premotor cortex	L	-57	5	40	7.34	0.022		0.0001
Postcentral gyrus	R	36	-31	43	6.70	0.042		0.0001
Thalamus	L	-15	-22	7	6.18	0.013		0.0001
Dorsal premotor cortex	R	27	-7	58	6.06	0.037		0.0001
Posterior cerebellum	R	18	-64	-44	5.63	0.026		0.0001
Putamen	L	-24	5	4	3.91		0.007	0.0001

P(FWE): statistical test whole-brain corrected for multiple comparison with threshold at the cluster level.

SVC(FWE): small volume correction (SVC) with sphere radius of 10 mm corrected for multiple comparisons with threshold at the cluster level.

P(unc.): statistical test whole-brain uncorrected.

Hemi: hemisphere – L (left), R (right).

-DLPFC: dorsolateral prefrontal cortex; SMA: supplementary motor area; PFC : prefrontal cortex.

Table S3. Neural activity during the action sequence execution period. Related to results in Figure 7.

Brain region	Hemi	Coordinates [x,y,z]			T-value	P (FWE)	SVC (FWE)	P (unc.)
**Subtraction Condition 1 > Condition 3 of results presented in Figure 7A**								
Superior frontal gyrus	R	21	2	67	5.56	0.047		0.000
Inferior parietal cortex	R	54	-40	55	4.74		0.024	0.000
Inferior parietal cortex	L	-66	-34	37	4.39		0.050	0.001
Precuneus	R	6	-55	58	4.15			0.001
Insula	R	33	20	7	3.89			0.001
Superior frontal gyrus	L	-21	2	67	3.86			0.001
Inferior frontal gyrus	R	51	14	13	3.80			0.001
Inferior frontal gyrus	L	-54	11	22	3.62			0.001
**Subtraction Condition 2 > Condition 3 of results presented in Figure 7B**								
Superior frontal gyrus	R	18	11	58	4.64	0.042		0.000
Inferior frontal gyrus	L	-33	35	10	4.42		0.011	0.001
Precuneus	L	-2	-64	58	4.41		0.011	0.001
Supramarginal	L	-63	-34	37	3.39		0.012	0.001
Supramarginal	R	54	-40	55	4.36		0.012	0.001
Anterior cingulate	R	12	32	13	4.29		0.014	0.001
Posterior cerebellum	R	33	-70	-47	4.19		0.018	0.001
DLPFC	R	27	32	31	4.10	0.021		0.000
Inferior operculum	L	-51	17	31	3.89		0.032	0.001
**Subtraction Condition 3 > (Condition 1 + Condition 2) of results presented in Figure 7C**								
Precentral gyrus	L	-33	-28	55	7.15	0.0001		0.0001
Posterior cingulate	L	-3	-40	28	5.83	0.0001		0.0001
Precuneus	L	12	-52	31	5.74		0.0001	0.0001
SMA	L	-6	-19	49	5.51		0.0001	0.0001
Insula	L	-39	-10	10	5.38	0.001		0.0001
Angular gyrus	R	57	-61	34	4.42		0.001	0.0001
Angular gyrus	L	-51	-64	37	5.32	0.005		0.0001
Insula	R	42	-1	16	4.97	0.006		0.0001
Putamen	L	-30	-7	-5	3.86		0.003	0.0001

P (FWE): statistical test whole-brain corrected for multiple comparison with threshold at the cluster level.

SVC (FWE): small volume correction (SVC) with sphere radius of 10 mm corrected for multiple comparisons with threshold at the cluster level.

P (unc.): statistical test whole-brain uncorrected.

Hemi: hemisphere – L (left), R (right).

-DLPFC: dorsolateral prefrontal cortex; SMA: supplementary motor area.

Table S4. Neural activity modulated by the reward score at the reward feedback period at the whole-brain level. Related to results in Figure 8.

Brain region	Hemi	Coordinates [x,y,z]			T-value	P (FWE)	SVC (FWE)	P (unc.)
Condition 1 immediate start trials								
Ventral putamen	R	15	5	-8	6.06	0.006		0.000
Caudate head	R	12	14	-2	5.28	0.006		0.000
Precuneus	R	6	-76	37	4.58		0.011	0.000
Inferior parietal cortex	L	-54	-61	43	4.32		0.005	0.000
Condition 2 immediate start trials								
External globus pallidus	L	-24	-13	-8	8.09	0.001		0.000
Hippocampus	L	-36	-19	-17	6.20	0.001		0.000
Parahippocampus	L	-18	-22	-17	5.33	0.001		0.000
Putamen	R	30	-13	-8	5.08		0.003	0.000
Hippocampus	R	39	-22	-14	3.98		0.019	0.001
Putamen	R	21	2	-8	4.53		0.003	0.000
Caudate head	R	12	20	1	4.19		0.009	0.000
Caudate head	L	-9	8	-2	4.58		0.003	0.000
Condition 1 delay start trials								
Caudate head	L	-6	14	-8	4.39	0.043		0.001
VMPFC	L	-9	26	-11	4.12		0.039	0.001
Ventral putamen	L	-18	2	-8	4.87		0.041	0.001
Condition 2 delay start trials								
Ventral putamen	R	27	8	-2	4.28	0.015		0.000
Caudate head	R	12	11	7	4.05		0.010	0.001

P (FWE): statistical test whole-brain corrected for multiple comparison with threshold at the cluster level.

SVC (FWE): small volume correction (SVC) with sphere radius of 10 mm corrected for multiple comparisons with threshold at the cluster level.

P (unc.): statistical test whole-brain uncorrected.

Hemi: hemisphere – L (left), R (right).

-DLPFC: dorsolateral prefrontal cortex; SMA: supplementary motor area.
